# Mouse Ataxin-2 Expansion Downregulates CamKII and Other Calcium Signaling Factors, Impairing Granule—Purkinje Neuron Synaptic Strength

**DOI:** 10.3390/ijms21186673

**Published:** 2020-09-12

**Authors:** Aleksandar Arsović, Melanie Vanessa Halbach, Júlia Canet-Pons, Dilhan Esen-Sehir, Claudia Döring, Florian Freudenberg, Nicoletta Czechowska, Kay Seidel, Stephan L. Baader, Suzana Gispert, Nesli-Ece Sen, Georg Auburger

**Affiliations:** 1 Experimental Neurology, Medical Faculty, Goethe University, Theodor Stern Kai 7, 60590 Frankfurt am Main, Germany; arsovicalexandar@gmail.com (A.A.); melanie.halbach@gmx.de (M.V.H.); jcanetpons@gmail.com (J.C.-P.); Gispert-Sanchez@em.uni-frankfurt.de (S.G.); 2 Department of Psychiatry, Psychosomatic Medicine and Psychotherapy, Medical Faculty, Goethe University, Heinrich-Hoffmann-Str. 10, 60528 Frankfurt am Main, Germany; dilhan.esen@kgu.de (D.E.-S.); Florian.Freudenberg@kgu.de (F.F.); 3 Faculty of Biosciences, Goethe-University, Max von Laue Strasse 9, 60438 Frankfurt am Main, Germany; 4 Dr. Senckenberg Institute of Pathology, Goethe University Frankfurt, Theodor Stern Kai 7, 60590 Frankfurt am Main, Germany; C.Doering@em.uni-frankfurt.de; 5 Institute of Anatomy, Anatomy and Cell Biology, University of Bonn, Nussallee 10, 53115 Bonn, Germany; nico.czechowska@uni-bonn.de (N.C.); kay_seidel@gmx.de (K.S.); sbaader@uni-bonn.de (S.L.B.)

**Keywords:** amyotrophic lateral sclerosis (ALS), fronto-temporal-lobar-dementia, tauopathies, synaptic plasticity, long-term potentiation, spatial learning, inositol signaling, neurexin, K-homology RNA-binding domain, fragile-X-associated tremor-ataxia syndrome

## Abstract

Spinocerebellar ataxia type 2 (SCA2) is caused by polyglutamine expansion in Ataxin-2 (ATXN2). This factor binds RNA/proteins to modify metabolism after stress, and to control calcium (Ca^2+^) homeostasis after stimuli. Cerebellar ataxias and corticospinal motor neuron degeneration are determined by gain/loss in ATXN2 function, so we aimed to identify key molecules in this atrophic process, as potential disease progression markers. Our *Atxn2*-CAG100-Knock-In mouse faithfully models features observed in patients at pre-onset, early and terminal stages. Here, its cerebellar global RNA profiling revealed downregulation of signaling cascades to precede motor deficits. Validation work at mRNA/protein level defined alterations that were independent of constant physiological ATXN2 functions, but specific for RNA/aggregation toxicity, and progressive across the short lifespan. The earliest changes were detected at three months among Ca^2+^ channels/transporters (*Itpr1*, *Ryr3*, *Atp2a2*, *Atp2a3*, *Trpc3*), IP_3_ metabolism (*Plcg1*, *Inpp5a*, *Itpka*), and Ca^2+^-Calmodulin dependent kinases (*Camk2a*, *Camk4*). CaMKIV–Sam68 control over alternative splicing of *Nrxn1*, an adhesion component of glutamatergic synapses between granule and Purkinje neurons, was found to be affected. Systematic screening of pre/post-synapse components, with dendrite morphology assessment, suggested early impairment of CamKIIα abundance together with the weakening of parallel fiber connectivity. These data reveal molecular changes due to ATXN2 pathology, primarily impacting excitability and communication.

## 1. Introduction

Spinocerebellar ataxia type 2 (SCA2) is an autosomal-dominantly inherited neurodegenerative disorder, caused by repeat expansion mutations in the ataxin-2 (ATXN2) poly-glutamine domain (polyQ, encoded by CAG repeats at the DNA level) [1,2,3]. Healthy individuals most commonly have 22 repeats, while the expansion of this domain beyond 33 repeats leads to the manifestation of SCA2. At presymptomatic and initial disease stages, muscle cramps (usually due to the dysregulation of cellular calcium homeostasis, treated with magnesium), hyporeflexia, progressive cerebellar ataxia, dysarthria and oculomotor deficits appear, in particular in abnormally slow saccades [4,5,6,7]. SCA2 usually manifests in the fourth decade of life, although the age of disease onset depends on the repeat size, genetic background and environmental factors. Longer expansion sizes lead to earlier manifestation and faster progression of the disease [8]. Intermediate size expansions between 27–33 repeats, often with the preservation of one CAA interruption within the otherwise pure CAG repeat, were reported to increase the risk of developing other neurodegenerative disorders, such as amyotrophic lateral sclerosis (ALS), Parkinson’s disease (PD) and Parkinsonism-plus syndromes [9,10,11,12].

*ATXN2* mutations affect many central nervous system regions, with pronounced atrophy of the spinal cord and cerebellum [13,14,15,16,17,18,19,20]. The basis of disease progression is the cumulative aggregation of mutant ATXN2 protein in the cytosol, with sequestration of its interaction partners into insolubility [21,22]. This phenomenon was documented to start very early in affected regions preceding the first appearance of disease signs [23]. The motor neurons degenerate at particularly early stages, with cortico-spinal projections progressively failing to excite the peripheral motor neurons via the neurotransmitter glutamate. This can be detected by electrophysiological analysis years before SCA2 patients will notice their first deficits of balance [24]. Within the cerebellum, the large Purkinje neurons are most conspicuous in their degeneration, and are the main site of pathology in various other ataxia disorders, as well. They receive excitatory glutamatergic input from cerebellar granule neurons via billions of parallel fibers with weak synaptic strength into the periphery of their dendrites, which can be potentiated or counterweighed by the glutamatergic input from brainstem inferior olivary neurons via climbing fibers, exerting strong synaptic input onto each Purkinje soma and their proximal dendrites. Throughout the postsynaptic spines, dendritic branches and cell body until the axon hillock, Purkinje neurons depend on Ca^2+^ fluxes to integrate stimuli and to trigger neural conductance, so these cells are characterized by their high abundance of various Ca^2+^ binding proteins [25]. The glutamatergic synapse between granule and Purkinje neurons was shown to play a central role in the pathogenesis of many spinocerebellar ataxia subtypes [26,27,28]. Accompanying the progressive morphological alterations of Purkinje neurons, granule cells are also affected in SCA2 cerebella upon detailed post-mortem histological analyses [29,30].

The ATXN2 protein has a large size of around 150 kDa in mouse, but is evolutionarily conserved in all eukaryotes, including yeast and plants. The N-terminal Like-Sm (Lsm) and Lsm-associated (Lsm-AD) domains maintain direct mRNA and miRNA binding, whereas the C-terminal PABP-associated motif (PAM2) associates ATXN2 indirectly to mRNA, by mediating its interaction with poly(A)-binding protein (PABP). Interspersed proline-rich domains (PRDs) modulate association with the endocytosis machinery [31], and the N-terminal polyQ domain has no specific function identified so far, other than being involved in neurological disease and facilitating interactions with other polyQ-containing proteins in disease [32]. Mainly localized in the cytoplasm, ATXN2 regulates cytosolic RNA processing and ribosomal translation, especially on the rough endoplasmic reticulum (rER) for proteins in the secretion pathway (which are subject to quality control and elimination via ER-associated degradation), while modulating growth signaling via receptor tyrosine kinase internalization at the plasma membrane [31,33,34,35]. Under various stress stimuli, ATXN2 localizes to ribonucleoprotein complexes known as stress granules (SGs), where it interacts with other RNA-binding proteins (RBPs) and several eukaryotic translation initiation factors (eIFs) to regulate mRNA stability and protein synthesis dynamics for survival [36,37,38,39]. Notably, altered SG dissociation dynamics due to intermediate-length *ATXN2* expansions underlie its pathogenic involvement in ALS. Important ALS-associated proteins, such as TDP-43, FUS and TIA-1, all of which are normally nuclear RBPs involved in RNA splicing, surveillance and triage, are sequestrated in the cytosolic SGs, where they are differentially modified and depleted from their native site of action [9,40,41,42,43]. Being a stress response protein itself, both transcript and protein levels of ATXN2 are induced by stressors, especially in cell types that are more susceptible to extracellular metabolic cues, such as neurons, glia, blood platelets, hepatocytes and pancreatic islet cells [44,45,46,47,48]. Increased amounts and activation of ATXN2 under stress by phosphorylation cascades were shown to suppress mTORC1 activity, due to sequestration of its components into SGs for energetic sustainability [34,49,50]. Global transcriptome and metabolome profiling of spinal cord tissue from two SCA2 mouse models highlighted a pronounced effect of ATXN2 and its sequence homolog ATXN2L on cholesterol and membrane lipid homeostasis [46,51,52,53]. Indeed, the loss of ATXN2 function in mouse leads to a metabolic excess syndrome manifested as diabetes mellitus with insulin resistance, lipid droplet accumulation in the liver, and hypercholesterolemia [48], whereas the loss of ATXN2L leads to mid-gestation embryonic lethality [53]. Mitochondrial protein import, tricarboxylic acid cycle and nutrient catabolism pathways are severely affected by ATXN2 loss across species [36,47,54]. Quite interestingly, depletion of ATXN2 expression by antisense oligonucleotides (ASOs) has shown massive benefit as a therapeutic intervention for ALS in mice and SCA2 patients [55,56].

As a recently generated and characterized model of ATXN2 pathology, the *Atxn2*-CAG100-KIN (KIN) mouse faithfully mirrors the spatio-temporal pattern of SCA2 neurodegeneration. It has been thoroughly examined regarding molecular features of mutant ATXN2, progression of pathology at the molecular and behavioral levels, and manifestation of disease signs. Initial immunohistochemistry analyses and in vivo nuclear magnetic resonance measurements revealed an overall atrophy of the brain and widespread aggregation of the mutant ATXN2 protein, rather than pure Purkinje cell pathology, as in transgenic models [23]. Thus, this new model has proven itself useful in dissecting not only Purkinje-specific aspects of disease, but also the contribution from other neuron populations and glia cell types, whose collective impact is being much better appreciated today in neurological disorders. In *Atxn2*-CAG100-KIN mice with homozygous expansion, behavioral signs of motor dysfunction appear at the age of six months (six mo), and deteriorate progressively, until the animals have to be sacrificed at the age of 14 mo when weight declines rapidly [23]. We showed previously that the CAG-repeat expansion within the DNA and mRNA of *Atxn2* leads to a chronic reduction of transcript and protein steady-state levels for Ataxin-2 already in embryonic cells, thus triggering an initial partial loss-of-function phenotype [23]. Through this mechanism, female homozygous KIN animals show an increased weight phenotype at the age of three mo, resembling *Atxn2*-KO animals [23]. However, the polyQ expansion within the protein triggers progressive accumulation of ATXN2 within cytosolic aggregates, leading to neurotoxicity via gain-of-function and corresponding phenotypes with time. At the molecular level, neuronal inclusion bodies and progressive expression dysregulations become detectable already at three mo, earlier than the first appearance of disease signs [23]. Given that any future neuroprotective therapy will be administered at this early disease stage, we sought to understand which molecular changes reflect this congenital partial loss-of-function of ATXN2 versus the progressive gain-of-toxic-function due to the cumulative aggregation process. For an initial unbiased survey of expression dysregulations, high-throughput transcriptome screening by microarrays was performed to compare *Atxn2*-CAG100-KIN cerebella with age- and sex-matched wild-type (WT) samples at three mo.

Using cerebellar homogenates from this genuine SCA2 animal model, we can be confident that the extracted mRNA has extremely little contribution from the brainstem-originating climbing fibers, so any glutamatergic dysregulations will reflect pathology in the parallel fiber synapses onto Purkinje spines. Altogether, our novel data suggest a simultaneous degeneration model for the SCA2 cerebellum, where granule layer affection starts in parallel to that of Purkinje layer at the pre-onset phase. The signaling cascades and synaptic components we investigated here represent excellent therapeutic targets, as there are readily available drugs targeting them, mostly in use for other neuronal maladies.

## 2. Results

### 2.1. Transcriptome Profile of Atxn2-CAG100-KIN Mouse Cerebellum before Disease Onset

**Figure 1:**
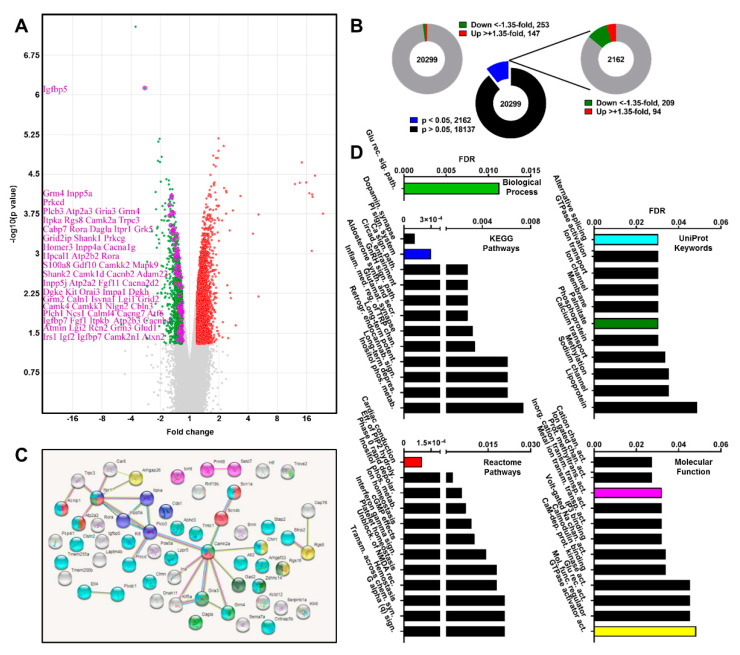
Global transcriptome profile of *Atxn2*-CAG100-KIN mouse cerebellum at pre-onset stage. (**A**) Volcano plot analysis of all the quantified mRNAs showing significant up- or downregulations beyond 20% fold-change in red and green, respectively. Highly significant and cerebellar pathology-relevant transcripts among downregulations are colored in magenta. Transcriptome analysis was performed with Affymetrix Clariom D oligonucleotide microarray technology, comparing 3 *Atxn2*-CAG100-KIN cerebellum samples with 3 age- and sex-matched WT controls. Statistical assessment of the expression data was done using the Affymetrix Transcriptome Analysis Console; (**B**) Representative diagrams depicting the numbers of all mRNA transcripts measured (20,299), versus those that are dysregulated more than 35%, regardless of statistical significance (253 down, 147 up), with the number of significant alterations among all (2162), and the numbers of nominally significant (*p* < 0.05) dysregulations in either direction more than 35% (209 down, 94 up); (**C**) Protein interaction network of the significantly downregulated transcripts (<−1.35 fold) generated with STRING database (https://string-db.org/) revealed a core network with two central nodes of *Itpr1* and *Camk2a*. Many factors in the network contributed to significantly altered pathways and cellular processes identified by Functional Enrichment Analysis function of STRING, and are highlighted in different colors corresponding to the colored bars in the subsequent panel D; (**D**) Functional Enrichment Analysis results of STRING utilizing GO terms Biological Process, KEGG Pathways, Reactome Pathways, UniProt Keywords and GO terms Molecular Function. Top ten dysregulated pathways and cellular processes are depicted per database for clarity, and complete lists are available in Supplementary Table S2. Colored bars represent pathways corresponding to similarly colored proteins in panel C.

More than 60,000 oligonucleotide spots were quantified, identifying 20,299 coding mRNAs, 1729 miRNA precursors, 130 miRNAs, 107 small RNAs (plus 2621 non-identified), 16 mitochondrial tRNAs, 388 ascertained or predicted ribosomal RNAs, 2758 pseudogenes, 24 unassigned and 31,717 non-coding transcripts (Supplementary Table S1). There was no preferential impact of ATXN2 expansion on one of these RNA classes. Our main focus in this study was on more than 20,000 coding mRNAs to elucidate disease pathology. Considering that the dataset comes from an age before the onset of motor deficits, and that the relevant dysregulations will not be massive at this stage, we empirically set a low 35% expression dysregulation threshold for data filtering, as already successfully done for Parkinson’s disease [57], instead of the commonly used 50% threshold. Among all quantified mRNA oligonucleotides, 2162 showed a significant expression change, revealing 209 significantly downregulated (< 65%) and 94 significantly upregulated (> 135%) transcripts (Figure 1A,B). The upregulated transcript group was dominated by the alteration of some of the hundreds of olfactory and vomeronasal receptors, whose number reflects the importance of smell for rodents, but are irrelevant for SCA2 patients. Among the significantly downregulated transcripts, insulin signaling mediator *Igfbp5*, cell migration and adhesion factor *Sema7a*, protein methyltransferases (*Prmt8*, *Icmt*), G-protein coupled receptor signaling mediator *Rgs8*, neurotransmitter receptors (*Gria3, Grm4*), and many Ca^2+^ signaling associated factors (*Plcb3*, *Inpp5a*, *Itpka*, *Itpr1*, *Atp2a3*, *Cabp7*, *Car8*, *Prkcd*, *Camk2a*, *Rora*) were prominent. Hence, we focused on the downregulations of the latter big group, and further examined the data with STRING database (https://string-db.org/) to generate a protein interaction network and assess the significant pathway enrichments. Interaction analysis (Figure 1C) revealed a tightly interconnected network with two rather central nodes at *Camk2a* and *Itpr1*. Many other Ca^2+^ signaling associated factors, Ca^2+^ transport machinery, ion channels and neurotransmitter receptors completed the network. Functional enrichment analysis by STRING in Biological Process, KEGG Pathways, Reactome Pathways, UniProt Keywords and Molecular Function databases revealed numerous significantly dysregulated subcellular processes mostly associated with Ca^2+^ mediated intracellular cascades, synaptic transmission and alternative splicing (Figure 1D).

In summary, the global transcriptome profile of *Atxn2*-CAG100-KIN cerebellum at pre-onset stage revealed a collective downregulation of various Ca^2+^-associated factors involved in its transport or downstream intracellular signaling cascades. These findings are in line with the well-established involvement of Ca^2+^ in various cerebellar maladies through altered neuronal excitability [58].

### 2.2. Ataxin-2 Pathology Alters the Expression of Ca^2+^ Channels and Transporters

We aimed to validate the high-throughput transcriptome data, focusing on Ca^2+^-associated transcripts and their role in ATXN2 expansion-driven disease and in complete ATXN2 loss. This work also extends previous findings that report similar dysregulations of Ca^2+^-associated transcripts in other mouse mutants [59,60]. Targeted expression analyses of the most relevant candidates were performed by reverse-transcriptase quantitative polymerase chain reaction (RT-qPCR). Protein abundance was assessed for promising factors, where specific and sensitive commercial antibodies were available. The levels of various Ca^2+^ channels and transporters were quantified in *Atxn2*-CAG100-KIN cerebella at different stages of disease progression (3 and 14 mo) and were also tested in *Atxn2*-KO cerebella, in order to distinguish partial loss-of-function effects from progressive aggregation pathology.

**Figure 2:**
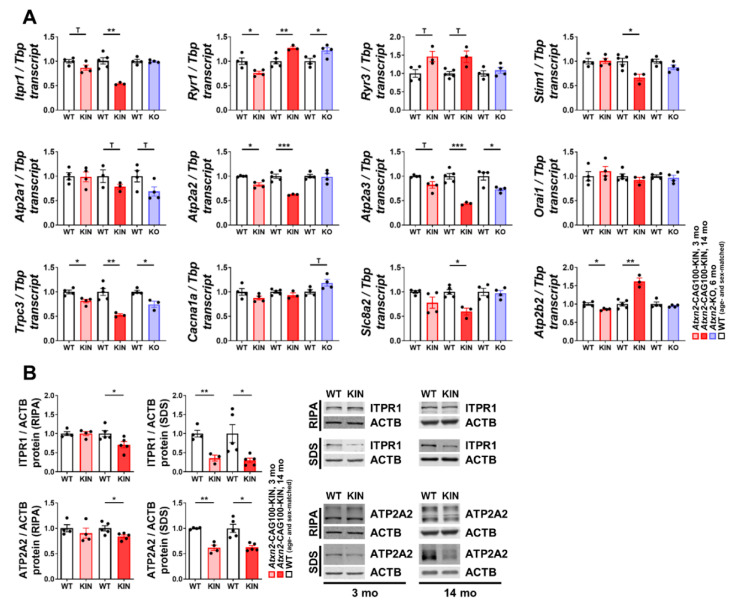
Expression analyses of Ca^2+^ channels, transporters and associated factors in *Atxn2*-CAG100-KIN cerebellum throughout the disease course. (**A**) Transcript levels of various plasma membrane and ER resident Ca^2+^-associated factors in *Atxn2*-KO (blue bars) and *Atxn2*-CAG100-KIN versus WT mouse cerebellum at pre-onset stage of 3 mo (pink bars) and terminal stage of 14 mo (red bars) were measured by RT-qPCR; (**B**) Protein levels of ER resident Ca^2+^ channel ITPR1 and transporter ATP2A2 in soluble/cytosolic (RIPA) and insoluble/membrane-associated (SDS) protein fractions of *Atxn2*-CAG100-KIN mouse cerebellum at pre-onset and final disease stages were determined by quantitative immunoblots. Student’s *t*-test with Welch’s correction; 0.05 < *p* < 0.1 ^T^, *p* < 0.05 *, *p* < 0.01 **, *p* < 0.001 ***. Further information regarding individual fold changes and *p*-values can be found in Supplementary Table S3.

Depending on their expression profile at pre-onset and late disease stages, we categorized the transcript changes as “early” (present at 3 mo and persisting at 14 mo), and “late/secondary” changes (occurring at 14 mo). Early changes occurred for ER-membrane channels *Itpr1* (encoding the protein IP3R) and *Ryr3* (RYR), transporters *Atp2a2* (SERCA2) and *Atp2a3* (SERCA3), and plasma membrane channel *Trpc3* (TRPC3). In contrast, ER-membrane factors *Ryr1* (RYR), *Stim1* (STIM1), *Atp2a1* (SERCA1), plasma membrane carrier *Slc8a2* (NCX) and transporter *Atp2b2* (PMCA) showed rather late dysregulations in disease process (Figure 2A). Among the early and late changes, *Ryr1*, *Atp2a1*, *Atp2a3* and *Trpc3* also showed similar dysregulations in *Atxn2*-KO cerebellum, suggesting a physiological regulation of these transcripts by ATXN2 protein. Plasma membrane channels *Orai1* (ORAI1) and *Cacna1a* (CaV2.1) were found unaltered throughout the disease course or in *Atxn2*-KO cerebellum (Figure 2A). Two factors of special interest, ITPR1/IP3R and ATP2A2/SERCA2, were further analyzed at the protein level in cytosolic (extracted by RIPA buffer) and insoluble/membrane-associated (pellet subsequently treated with 2x SDS buffer) fractions of *Atxn2*-CAG100-KIN mouse cerebellum (Figure 2B). At the pre-onset stage, both proteins were unaltered in the cytosolic soluble protein fraction, and showed subtle reductions with disease progression at 14 mo. In the more insoluble protein fraction, ITPR1 showed a strong decrease at 3 mo, persisting throughout disease progression until the terminal stage of 14 mo (Figure 2B, individual fold changes and *p*-values are available in Supplementary Table S3). Interestingly, ITPR1 was previously reported to interact exclusively with expanded ATXN2, and to have increased abundance in the insoluble protein fraction of aged *Atxn2*-CAG42-KIN cerebellum [59]. Similar to ITPR1, ATP2A2 also showed decreased abundance in the insoluble protein fraction at both 3 mo and 14 mo of age (Figure 2B, Supplementary Table S3).

Overall, the expression data highlight *Itpr1, Atp2a2* and *Atp2a3* as the early-onset downregulations, which worsen over time in parallel to the expansion-driven aggregation process and are absent in *Atxn2*-KO cerebellum.

### 2.3. Subcellular Ca^2+^ Imbalance Promotes Ataxin-2 Relocalization into Stress Granules

**Figure 3:**
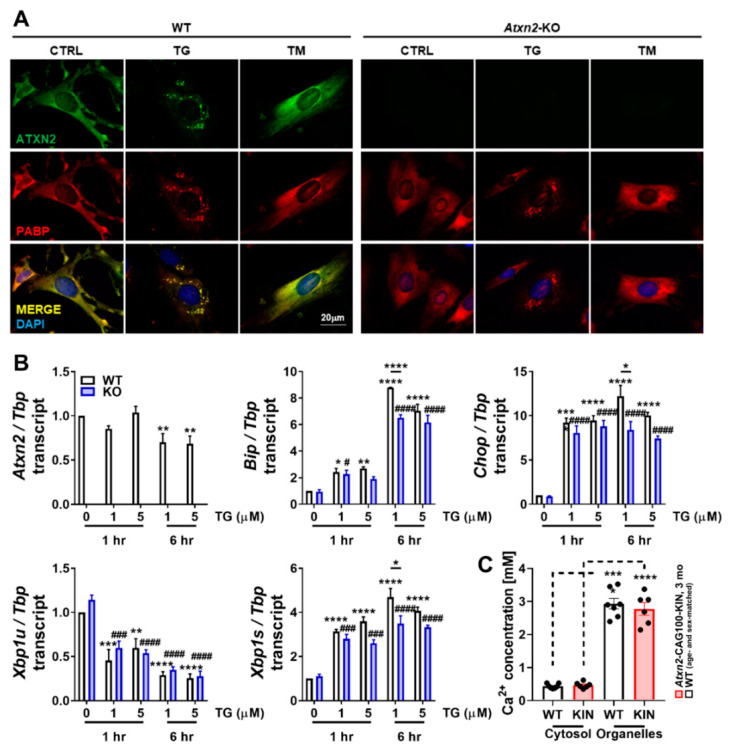
The effect of subcellular Ca^2+^ imbalance on ATXN2 localization and expression in mouse embryonal fibroblasts. (**A**) Immunocytochemical assessment of ATXN2 and SG marker PABP in WT and *Atxn2*-KO MEFs under thapsigargin-(TG, 5 μM, 6 h) or tunicamycin-induced (TM, 10 μg/mL, 6 h) ER stress. ATXN2 relocalization into PABP-positive SGs was observed, solely upon cytosolic Ca^2+^ imbalance driven by TG, but not upon blockage of N-glycosylation by TM. *Atxn2*-KO MEFs did not show a difference in SG formation upon TG treatment. (**B**) Transcriptional regulation of *Atxn2* and ER stress markers under TG treatment, in a time- and dosage-dependent setup. Three different clones of WT and *Atxn2*-KO MEF pairs were treated simultaneously with 1 μM or 5 μM TG for 1 h or 6 h. Stress response was already visible at 1 h for both TG dosages, and further increased at 6 h. While *Atxn2* showed a significant downregulation under TG treatment, ER stress markers *Bip*, *Chop* and *Xbp1s* showed a suppressed induction in the absence of ATXN2 in KO cells (blue bars). Expression data obtained by RT-qPCR; (**C**) Colorimetric Ca^2+^ concentration measurement in cytosolic and organelle-enriched fractions of WT and *Atxn2*-CAG100-KIN cerebellum at three mo of age. Higher Ca^2+^ concentrations were observed in the membrane-encapsulated organelle fraction with no difference between WT and KIN animals. Statistical assessment of the cell culture data was done using 2-way ANOVA with multiple testing corrections. Statistical assessment of the cerebellar Ca^2+^ measurement was done using Student’s *t*-test with Welch’s correction; *p* < 0.05 *, *p* < 0.01 **, *p* < 0.001 ***, *p* < 0.0001 ****. Hashtag (#) indicates comparison of KO cells with untreated KO cells, with *p* < 0.05 #, *p* < 0.001 ###, *p* < 0.0001 ####. Further information regarding individual fold changes and *p*-values can be found in Supplementary Table S3.

Potent dysregulations observed in various ER and plasma membrane associated Ca^2+^ factors at early disease stage could lead to the mislocalization of Ca^2+^ ions among the subcellular organelles. It is known that Ca^2+^ ions are deliberately kept at very high concentrations in the ER for proper chaperone function in the ER lumen and for structural integrity of the cytosolic proteins, which tend to expose their internal hydrophobic residues at high Ca^2+^ concentration and start forming aggregates [61]. Thus, it is intriguing to investigate whether an aggregation-prone protein such as ATXN2 is affected by high cytosolic Ca^2+^ concentration and changes its subcellular distribution or expression. In order to test this notion, we treated WT and *Atxn2*-KO primary mouse embryonal fibroblasts (MEFs) with thapsigargin (TG), an ER-stress inducer known to increase Ca^2+^ concentration in the cytosol by blocking the ATP2A2 (SERCA2) transporter [61]. As hypothesized, TG-mediated high cytosolic Ca^2+^ concentration triggered ATXN2 protein to redistribute into PABP-positive SGs (Figure 3A). The complete loss of ATXN2 did not alter SG dynamics under TG treatment, in agreement with the previous findings under sodium arsenite-driven oxidative stress [34]. In order to ensure that SG formation and ATXN2 relocalization are induced by increased cytosolic Ca^2+^ levels *per se*, and not due to ER-stress response in general, we performed tunicamycin (TM) treatment in parallel, which induces ER-stress via the blockage of N-linked protein glycosylation and accumulation of the un- or misfolded proteins in the ER lumen [61]. This arm of ER-stress induction did not lead to SG formation or any relocalization of ATXN2 (Figure 3A). In conclusion, the subcellular localization of ATXN2 was found to be specifically modulated by cytosolic Ca^2+^ levels, but not by ER stress in general.

As a stress-response factor, ATXN2 is often regulated transcriptionally under stress conditions, mostly towards upregulation [34,36]. Therefore, we analyzed the regulation of *Atxn2* expression under increased cytosolic Ca^2+^ concentration in a time- and dose-dependent manner. While no change was observed in *Atxn2* levels after 1 h of TG treatment, prolonged Ca^2+^ imbalance over 6 h led to a decrease in *Atxn2* abundance, independent of the drug dosage (Figure 3B), which is compatible with the notion of delayed protein turnover due to the aggregation of ATXN2 leading to diminished transcriptional resynthesis. The expression of ER stress markers was also quantified under TG treatment to validate successful stress induction and to observe the potential effects of ATXN2 loss in ER-stress response. ER lumen chaperone *Bip* and further downstream apoptosis promoting factor *Chop* were readily upregulated upon 1 h of TG treatment, with further inductions at 6 h (Figure 3B). Splicing of *Xbp1* transcript by ER membrane-associated RNase IRE1 is another hallmark of ER stress. Quantification of the unspliced isoform (*Xbp1u*) revealed a time-dependent decrease, while the spliced isoform (*Xbp1s*) showed a time-dependent increase in abundance under TG treatment (Figure 3B). Interestingly, lower dosage of TG at 6 h seemed to induce more of a stress response than the higher dosage suggested by *Bip*, *Chop* and *Xbp1s* levels in WT cells. Quantification of the same transcripts in *Atxn2*-KO MEFs revealed no initial dysregulation in untreated cells due to ATXN2 loss, although a subtle increase was observed in *Xbp1u* levels without significance. At 6 h under lower dosage of TG, *Bip*, *Chop* and *Xbp1s* levels showed a significant induction deficit in *Atxn2*-KO cells, suggesting that ATXN2 is necessary for a maximal ER stress response when Ca^2+^ homeostasis is abnormal.

Recent work on ATXN2 revealed its role in mitochondrial dynamics and proteostasis during stress through the modulation of PINK1 and OPA1 levels [45], regulation of mitochondrial protein import [54], and affecting metabolic enzymes involved in TCA cycle, amino acid and fatty acid catalysis [36,47]. In addition, ATXN2 was found to localize at the rER, and regulate two important ER-associated aspects: protein translation and Ca^2+^ homeostasis [37,59]. Therefore, we asked whether the contact sites between mitochondria and ER, namely mitochondria-associated membrane (MAM) complex, could be affected from ATXN2 expansion, and somehow initiate the pathology targeting two organelles at once. Expression analyses of important MAM complex components *Mcu*, *Micu1*, *Micu2*, *Micu3*, *Smdt1*, *Vdac1*, *Grp75*, *Mfn1*, *Mfn2* and *Sigmar1* in *Atxn2*-CAG100-KIN cerebellum revealed no major alteration of this complex, even at the terminal disease phase (Supplementary Figure S1A). Yet, subtle downregulations were observed for many components, which could downgrade the collective activity of the complex. The previously mentioned Ca^2+^ channel ITPR1 (Figure 2A,B) also belongs to the MAM complex. Thus, the biggest dysregulation among all MAM complex members at the terminal phase was found to occur for *Itpr1* with an early onset expansion-associated downregulation, while the subtle changes of the other members could represent a secondary coping mechanism.

Taking these findings into account, we questioned the extent of ER stress and associated transcript inductions in *Atxn2*-CAG100-KIN mouse cerebellum at terminal stage. Expression analyses of ER-resident primary unfolded protein response (UPR) mediators *Perk*, *Ire1* and *Atf6* interestingly revealed no transcriptional induction, but decreased abundance (Supplementary Figure S1B). Their luminal regulator *Bip*, and direct downstream effectors *Xbp1u* and *Xbp1s* were found unaltered. The further downstream effector *Chop* was found subtly upregulated, together with its transcriptional activator *Atf4*, which could have arisen from a plethora of stress inputs of different origins (Supplementary Figure S1B). This set of data indicates that, although ATXN2 might acutely modulate ER stress response and the induction of apoptosis, chronic UPR activation is not a prominent aspect of disease pathology in the brain, showing only mild alterations at the terminal stage when numerous stress stimuli converge and cross-activate each other.

Finally, after establishing the effect of cytosolic Ca^2+^ concentration on ATXN2 distribution, and the lack of significant ER stress induction at terminal stage, we aimed to see if there is overall subcellular Ca^2+^ mislocalization in the intact cerebellum at pre-onset stage. Fresh cerebella were fractionated into cytosolic and membrane-encapsulated organelle rich fragments, and Ca^2+^ concentrations of these lysates were measured with a commercially available colorimetric kit. As expected, organelle rich fraction showed a significantly higher Ca^2+^ content in comparison to cytosol in both WT and *Atxn2*-CAG100-KIN samples (Figure 3C). However, no change was observed by this steady-state assay in Ca^2+^ levels or subcellular distribution in KIN samples compared to WT animals, indicating that chronic Ca^2+^ mislocalization is not an initial cause of the disease, and that the early onset alterations of several ER, and plasma membrane channels and transporters are able to maintain the balance at this stage.

### 2.4. Pre-Onset Dysregulation of the Ca^2+^/Calmodulin-Dependent Protein Kinase Pathway

Aside from the role of Ca^2+^ in the maintenance of ER dynamics and proteostasis, it is also an important secondary messenger in signaling cascades, especially for excitable cells, such as neurons and myocytes. While low cytosolic concentrations normally prevail, bursts of Ca^2+^ increase, for instance, due to synaptic impulse, and are recognized as specific input, and activate the Ca^2+^/Calmodulin-dependent protein kinase (CaMK) pathway. CaMKs are involved in a broad spectrum of cellular processes, such as regulation of phosphorylation cascades, gene expression, mRNA splicing, metabolism and cell survival/death [62].

We observed several members of the CaMK family and associated pathway components among the significantly dysregulated transcripts in the global transcriptome data, such as *Camk2a*, *Camkk2*, *Gria3*, *Grm4*, *Plcb3*, *Inpp5a* and *Pcp4* (Supplementary Table S1). Targeted expression analyses of these transcripts together with other pathway components were performed in 3 mo and 14 mo *Atxn2*-CAG100-KIN cerebellum in parallel to *Atxn2*-KO samples. 

**Figure 4:**
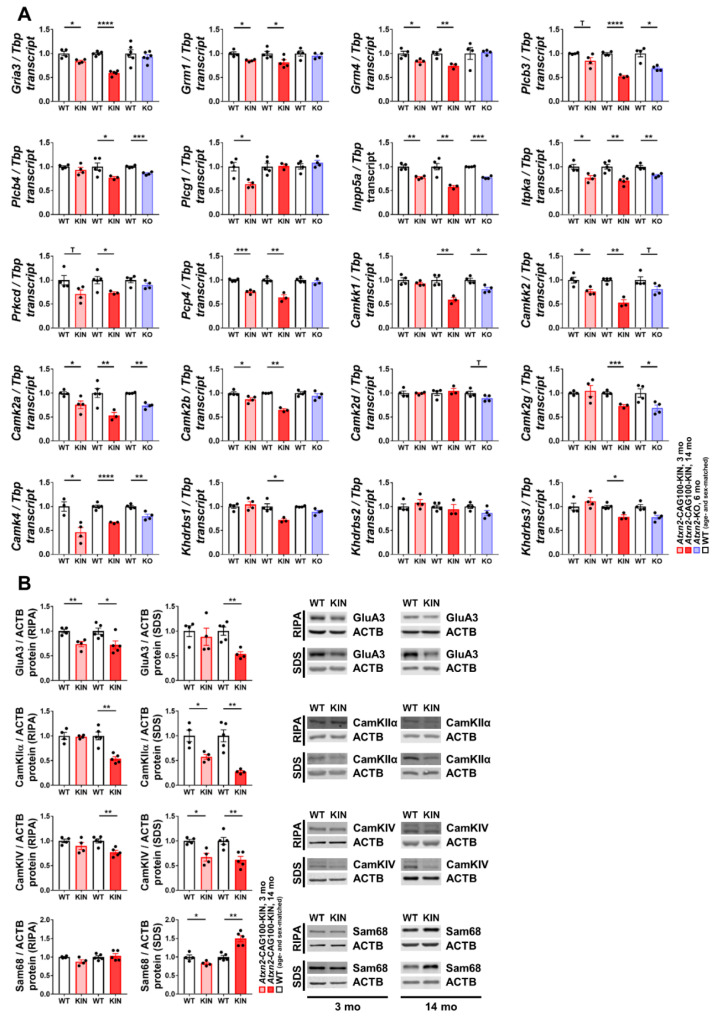
Expression analyses of Ca^2+^ associated subcellular signaling pathways in *Atxn2*-CAG100-KIN cerebellum throughout disease course. (**A**) Transcript levels of cytoplasmic IP_3_ metabolism components (*Gria3, Grm1*, *Grm4*, *Plcb3*, *Plcb4*, *Plcg1*, *Inpp5a, Itpka, Prkcd*), Ca^2+^-CaM signaling components (*Pcp4*, *Camkk1*, *Camkk2*, *Camk2a*, *Camk2b*, *Camk2d*, *Camk2g*, *Camk4*) and downstream CaMKIV targets (*Khdrbs1-3*) were quantified by RT-qPCR in *Atxn2*-KO (blue bars) and *Atxn2*-CAG100-KIN mouse cerebellum at pre-onset (pink bars) and terminal (red bars) disease stages; (**B**) Protein levels of GluA3, CaMKIIα, CaMKIV and Sam68 in soluble (RIPA) and insoluble (SDS) fractions of *Atxn2*-CAG100-KIN mouse cerebellum at pre-onset and final disease stages were determined by quantitative immunoblots. Student’s *t*-test with Welch’s correction; 0.05 < *p* < 0.1 ^T^, *p* < 0.05 *, *p* < 0.01 **, *p* < 0.001 ***, *p* < 0.0001 ****. Further information regarding individual fold changes and *p*-values can be found in Supplementary Table S3.

Upon extracellular activation of receptor tyrosine kinases (RTKs) or ionotropic glutamate receptors (e.g., GluAs) or metabotropic glutamate receptors (mGluRs), the phospholipase C (PLC) isoforms are activated at the intracellular plasma membrane leaflet and catalyze the cleavage of PIP_2_ (phosphatidylinositol-4,5-bisphosphate) into IP_3_ (inositol 1,4,5-trisphosphate), as well as DAG (diacylglycerol) [63,64]. IP_3_ may directly bind and activate IP3R leading to Ca^2+^ flux from ER to cytosol; alternatively, it can be phosphorylated by ITPKA or de-phosphorylated by INPP5A for recycling [65]. On the other hand, DAG signals either directly to plasma membrane Ca^2+^ channel TRPC3 or indirectly via activating protein kinase C (PKC) [63]. The ionotropic AMPA receptor subunit *Gria3* (encoding GluA3) showed an early and strongly progressive downregulation, and the two mGluR isoforms *Grm1* (mGluR1) and *Grm4* (mGluR4) showed a subtle yet significant downregulation in *Atxn2*-CAG100-KIN cerebella already at three mo of age (Figure 4A). In view of the relevance of *Gria3* dysregulation as an early risk marker and later progression marker, these changes were assessed at the protein level. At the pre-onset stage of three mo, GluA3 abundance showed a significant decrease to 74% in the soluble but not the insoluble/membrane-bound protein fraction. At the terminal stage of 14 mo, GluA3 abundance was similarly decreased in the soluble fraction (to 72%), but now also in the membrane-bound fraction to 53% (Figure 4B).

As important downstream membrane-associated signaling factors, the PLC isoforms β3 and β4 (*Plcb3* and *Plcb4*) also showed a subtle downregulation, which became more prominent later in disease pathology. The presence of a similar dysregulation of both transcripts in *Atxn2*-KO cerebellum indicates their potential modulation by native ATXN2 function (Figure 4A). PLC isoform γ1 (*Plcg1*) showed a significant downregulation at three mo of age, and interestingly, no alteration afterwards or in *Atxn2*-KO samples, which represents a very specific early-pathology marker. *Inpp5a* and *Itpka* expression were found significantly downregulated at pre-onset phase, progressively decreasing with disease pathology at 14 mo, and diminished in *Atxn2*-KO cerebella, representing ATXN2 native function-dependent progression markers. Downstream of DAG, however, PKC subunit δ (*Prkcd*) showed an expansion pathology-specific downregulation in *Atxn2*-CAG100-KIN cerebella, starting from pre-onset stage (Figure 4A). This arm of the signaling pathway revealed that the polyQ expansion influence starts from the plasma membrane with reduced glutamate receptor transcript levels, extends to altered conversion of membrane lipids to DAG and IP_3_, and reaches IP_3_ recycling that is probably diminished.

During further signal transduction in the cytoplasm, CaMK is activated upon Calmodulin (CaM) association with four Ca^2+^ ions. PCP4 (or PEP-19) was shown to be a regulator of Ca^2+^/CaM association and dissociation dynamics [66]. Activated CaM further initiates a phosphorylation cascade involving CaMK-kinases (CaMKKs), CaMKs and downstream targets. In *Atxn2*-CAG100-KIN cerebellum, *Pcp4* showed a prominent downregulation, starting early and progressing with the disease. Lack of its dysregulation in *Atxn2*-KO cerebellum indicates an expansion-driven alteration of *Pcp4* expression (Figure 4A). Both CaMKKs, *Camkk1* and *Camkk2*, showed an ATXN2-dependent downregulation to 80% in *Atxn2*-KO cerebellum, and a decrease to 60% at the late phase of the disease. In addition, *Camkk2* showed a significant downregulation at the pre-onset phase, showing an earlier alteration than *Camkk1* (Figure 4A). Due to their thoroughly investigated roles in dendritic spine morphology and synaptic integrity maintenance, we further examined the dysregulations in CaMKII isoforms and CaMKIV expression. Both *Camk2a* and *Camk2b* isoforms showed a progressive decrease in expression parallel to the disease pathology, with *Camk2a* also showing a mild downregulation in *Atxn2*-KO cerebellum. *Camk2g* showed downregulation later in disease and *Atxn2*-KO cerebellum, whereas no significant change was observed in *Camk2d* levels at any age or in *Atxn2*-KO (Figure 4A). Similar to the expression profile of *Camk2a*, *Camk4* also showed prominent downregulation at the pre-onset phase, maintained throughout the disease until the terminal phase, and also mildly downregulated in *Atxn2*-KO cerebellum (Figure 4A). Both transcripts represent potent markers of early pathology and disease progression, and were therefore also validated at the protein level. At the pre-onset stage of 3 mo, CaMKIIα showed no dysregulation in the soluble protein fraction, but showed a significant downregulation to 57% in the insoluble/membrane-bound protein fraction. At the terminal stage of 14 mo, CaMKIIα abundance had progressively decreased in both fractions (Figure 4B). Similarly, CaMKIV abundance was unaltered at the pre-onset phase in soluble fraction, with a significant decrease to 77% in the insoluble fraction. At 14 mo, CaMKIV protein was also found significantly downregulated in both fractions, a decrease in parallel to CaMKIIα and disease progression (Figure 4B).

Overall, these data suggest that ATXN2 loss modulates intracellular Ca^2+^ signaling component levels, especially that of PLCβ and IP_3_ recycling enzymes, but does not alter glutamate receptor levels (as indicated by mRNA changes in *Atxn2*-KO cerebellum). The ATXN2 expansion at 14 mo triggers a stronger dysregulation in the same direction, when compared to the *Atxn2*-KO, for these Ca^2+^ signaling components (namely *Plcb3*, *Plcb4*, *Inpp4a*, *Itpka*, *Camkk1*, *Camk2a*, *Camk4*), and in addition, it affects the ionotropic and metabotropic glutamate receptors.

### 2.5. Impact of Ataxin-2 Pathology on the CamKIV-Modulated RNA Splicing Factor Khdrbs1/Sam68

Initiation of Ca^2+^/CaM-dependent signaling cascade in cerebellar granule neurons upon frequent stimulation activates CaMKIV and leads to the phosphorylation of its substrates, one of which is the K-homology domain, containing RNA-binding protein Sam68 (gene symbol *Khdrbs1*). Phosphorylated Sam68 detaches from its target mRNAs, thus differentially regulating their alternative splicing [67]. Thus, any loss of CaMKIV-dependent phosphorylation might influence the nuclear distribution of Sam68, and its association with ribonucleoprotein (RNP) granules, known for their poor solubility in phase separation. One of the Sam68 target transcripts is *Nrxn1* pre-mRNA, encoding different isoforms of the pre-synaptic Neurexin-1 protein involved in structural synapse maintenance [68]. Quantitative assessment of Sam68 (*Khdrbs1*) expression revealed an expansion-specific downregulation at late disease stage in *Atxn2*-CAG100-KIN cerebellum, without an alteration at the pre-onset stage or in *Atxn2*-KO (Figure 4A). Likewise, the two closely related family members of Sam68, namely Slm1 and Slm2 (encoded by *Khdrbs2* and *Khdrbs3*, respectively), also showed no dysregulation in *Atxn2*-KO cerebella. Only Slm2 (*Khdrbs3*) showed an expansion-driven downregulation at the terminal disease stage, while Slm1 (*Khdrbs2*) showed no dysregulation at the transcript level throughout the disease course (Figure 4A).

At the protein level, Sam68 abundance in the soluble fraction showed no alteration at the early or late disease stages. However, in the SDS fraction that is enriched for insoluble cytosolic aggregates or membrane encapsulated organelles including nuclei, Sam68 abundance was found to be subtly decreased at the age of three mo, but significantly increased up to 150% at the terminal stage of 14 mo (Figure 4B). This observation is consistent with the normally nuclear localization of Sam68, its potential redistribution into RNP granules and potential interaction with ATXN2. We therefore questioned whether Sam68 could be trapped in cytosolic ATXN2 aggregates like many other nuclear RNA processing proteins such as TDP-43, FUS, TIA-1 [52,69], which would explain its increased abundance at the terminal stage. Immunohistochemical staining of Sam68 and ATXN2 confirmed the nuclear localization of Sam68 in Purkinje and granule neurons of the WT cerebellum at 14 mo of age, while ATXN2 showed a diffuse cytosolic localization (Supplementary Figure S2). In the Purkinje neurons of *Atxn2*-CAG100-KIN cerebellum, ATXN2 was found clumped into a single large aggregate localized at the entrance of the dendritic arbor, in agreement with a previous report [23]. Sam68 immunostaining in *Atxn2*-CAG100-KIN cerebellum did not seem to co-localize with these aggregates and was solely detected in the nuclei (Supplementary Figure S2). However, the nuclear Sam68 signal in both Purkinje and granule neurons of *Atxn2*-CAG100-KIN cerebellum was stronger and more punctate compared to WT (Supplementary Figure S2), indicating the higher nuclear abundance of Sam68, which explains its upregulation in the SDS fraction at this age.

In short, detailed investigation of the glutamate-dependent synaptic signaling and Ca^2+^/CaM-dependent kinase signaling revealed an early defect in Ca^2+^ association dynamics of CaM, and reduced the expression of many cascade components at both transcript and protein levels, including GluA3, CaMKIIα and CaMKIV as early risk and progression markers of the disease. Sam68, a downstream target of CaMKIV, was found to be affected later in disease progression with increased nuclear abundance and granular redistribution, but without detectable sequestration into cytosolic ATXN2 aggregates.

### 2.6. Alternative Splicing Profile of Nrxn1 in Ataxin-2 Pathology

**Figure 5:**
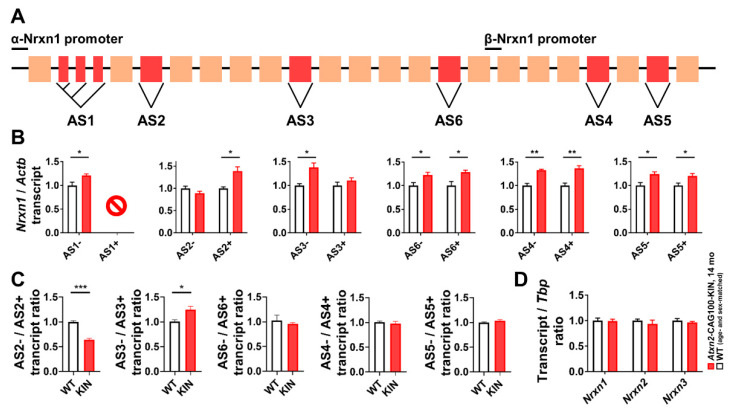
Expression analyses of *Nrxn1-3* transcripts and alternative splicing isoforms of *Nrxn1* in *Atxn2*-CAG100-KIN cerebellum at 14 mo. (**A**) Schematic representation of *Nrxn1* structure showing six alternative splice (AS) sites. Constitutive exons are depicted in beige and alternatively spliced exons are in red. The transcript structure and spatial distribution of the AS sites were adapted from Treutlein et al. 2014 [70]; (**B**) Transcript levels of spliced (−) or unspliced (+) variants of *Nrxn1* at AS1-6 show altered splicing in *Atxn2*-CAG100-KIN cerebellum at 14 mo. Site-specific primers were designed to selectively amplify exon inclusion or excision at a given AS site by RT-qPCR. At AS1 site, only AS1(−) isoform lacking all intermediate exons could be quantified with this method, due to the structural complexity of the region and impossibility of proper primer design for all splice variants; (**C**) Splicing activity ratio at AS2-6 sites of *Nrxn1* reveal the missplicing of AS2 and AS3 sites in *Atxn2*-CAG100-KIN cerebellum at 14 mo. The ratio between spliced (−) to unspliced (+) variants shown in panel B was calculated for each AS site to assess splicing activity at each site. Significantly decreased activity at AS2, and significantly increased splicing at AS3 was observed; (**D**) Total levels of *Nrxn1-3* transcripts measured by RT-qPCR amplifying constitutive exons showed no dysregulation in *Atxn2*-CAG100-KIN cerebellum at the terminal disease stage. Student’s *t*-test with Welch’s correction; *p* < 0.05 *, *p* < 0.01 **, *p* < 0.001 ***. Further information regarding individual fold changes and *p*-values can be found in Supplementary Table S3.

It is well known that expanded ATXN2 interacts with other RNA-binding proteins, and prominently with the splice modulator TDP-43. To investigate the potential downstream effects of CaMKIV-Sam68 pathway alterations observed in *Atxn2*-CAG100-KIN mouse cerebellum starting before disease onset, we focused on the alternative splicing of Neurexin-1 (encoded by *Nrxn1*), a well-studied structural synapse component. *Nrxn1* transcript has six alternative splice (AS) sites, AS1-6 (Figure 5A), generating a plethora of mature mRNA and protein isoforms exhibiting differential interactions with synaptic cleft or post-synaptic membrane proteins, such as cerebellins, neuroligins, leucine-rich repeat proteins (LRRTMs), dystroglycan and latrophilins [68,70]. The neuronal activity-dependent splicing of *Nrxn1*, especially at AS4, is governed by Sam68, Slm1 or Slm2 in distinct neuronal populations [71]. In cerebellar granule neurons, Sam68 is the dominant regulator, and enhances the *Nrxn1* splicing towards AS4(−) isoform lacking the alternatively spliced exon at this site. Loss of Sam68 activity in cerebellum, or that of Slm1 and Slm2 in different CNS regions, has been shown to significantly diminish splicing activity at this site, therefore reducing the abundance of AS4(−) and increasing AS4(+) isoform [67,72,73]. In parallel to the widely studied AS4 site, we investigated the splicing activity at all *Nrxn1* AS sites in *Atxn2*-CAG100-KIN cerebellum at terminal disease stage, when *Khdrbs1* (Sam68) and *Khdrbs3* (Slm2) are significantly decreased.

Excision of the alternatively spliced exon at each AS site produces the “spliced” or (−) isoform, whereas retaining the exon generates the “unspliced” or (+) isoform. Due to high structural complexity of the AS1 site, only the AS1(−) variant lacking all intermediate exons was successfully quantified with RT-qPCR, showing a significant increase in *Atxn2*-CAG100-KIN cerebellum compared to WT animals, indicative of higher exon excision rate at AS1 (Figure 5B). While the AS2(−) isoform showed no significant alteration, AS2(+) isoform was found increased in *Atxn2*-CAG100-KIN animals indicating a tendency towards exon retention at this site. In contrast, exon excision at AS3 site was found to be increased, as supported by significantly high abundance of AS3(−) isoform in *Atxn2*-CAG100-KIN samples, without a change in AS3(+) levels. All the other splice sites located downstream, namely AS4, AS5 and AS6, showed the increased abundance of all splice variants in *Atxn2*-CAG100-KIN cerebellum, without a selective preference for (+) or (−) isoforms (Figure 5B). The ratio of spliced-to-unspliced variant abundance for each AS site reveals the splicing activity (i.e., exon excision rate) at this region. The assessment of excision rates at AS2-6 revealed significantly lower splicing activity at AS2 and significantly higher activity at AS3 in *Atxn2*-CAG100-KIN cerebellum at terminal disease stage, whereas no alteration of splicing rate was observed at AS4-6 (Figure 5C). The AS2(−) isoform of Nrxn1 protein was reported to selectively interact with the postsynaptic dystroglycan/dystrophin complex [74,75], and currently there are no studies revealing a selective interaction of Nrxn1 based on AS3 site splicing. Quantification of the total levels of *Nrxn1*, together with *Nrxn2* and *Nrxn3*, revealed no alteration in *Atxn2*-CAG100-KIN cerebellum at terminal disease stage (Figure 5D). 

Altogether, these results suggest synaptic connections between granule and Purkinje neurons in *Atxn2*-CAG100-KIN cerebellum to be affected by abnormal splicing of selective *Nrxn1* isoforms, rather than by the steady-state abundance change of all Neurexins.

Investigation of total *Nrxn1* level and AS4 splicing pattern, as the most studied *Nrxn1* splicing event, in *Atxn2*-KO and pre-onset *Atxn2*-CAG100-KIN cerebella showed no significant dysregulations (Supplementary Figure S3A), indicating that the splicing alterations emerge and contribute to disease pathogenesis in an expansion-driven manner and rather late in progression.

### 2.7. Morphological Assessment of Purkinje Cell Spines

**Figure 6:**
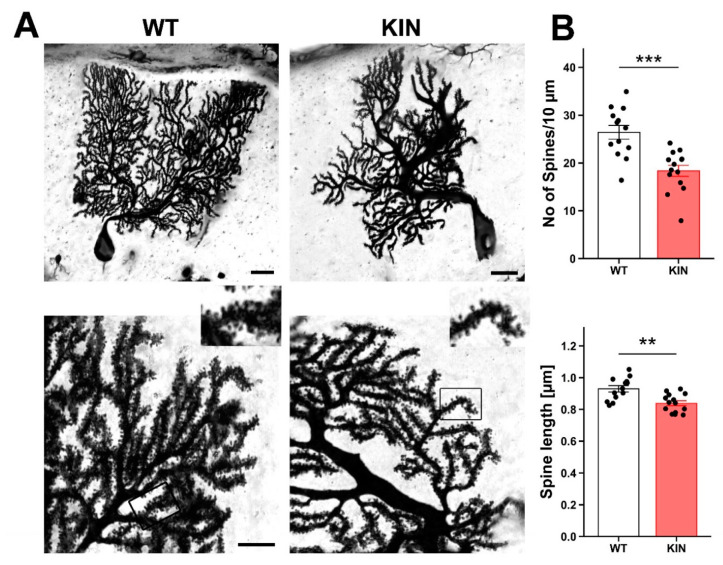
Golgi impregnation of Purkinje cells in WT and *Atxn2*-CAG100-KIN cerebella at pre-terminal age of 9 mo. (**A**) Representative images of Purkinje neurons of both genotypes in low and high magnifications. Scale bars indicate 20 µm (upper row) and 10 µm (lower row); in order to be able to appreciate differences in spine length and spine density, an inlet was added showing a dendritic segment at higher magnification. This image is a digital zoom-in at a factor of 2. It can be clearly seen that the density is much less in KIN mice, while the spine length is a little smaller. (**B**) Significant reductions of spine number and length were observed in *Atxn2*-CAG100-KIN Purkinje dendrites compared to WT (*n* = 13 WT vs. 14 KIN Purkinje cells, from 4 WT vs. 3 KIN animals). Levene’s test was used for evaluating equal data distribution, and ANOVA equals *t*-test was used to compare WT vs. KIN cells; *p* < 0.01 **, *p* < 0.001 ***. Further information regarding individual fold changes and *p*-values can be found in Supplementary Table S3.

Having established the early influence of ATXN2 expansion on CaMKIIα and CaMKIV signaling, and its downstream effects on the alternative splicing of presynaptic Neurexin-1 protein, we assessed whether the deficit of these crucial factors for synaptic plasticity and adhesion triggered morphological correlates for the synapses between granule and Purkinje neurons. Golgi silver impregnation of *Atxn2*-CAG100-KIN cerebella showed a significant reduction in spine length and spine density at the age of 9 mo, at a stage when motor incoordination had become apparent in each individual mouse (Figure 6). 

Overall, both poles of the parallel fiber synapse onto Purkinje spines were found to be influenced by the ATXN2 expansion; this was prominently reflected by CaMKIV-dependent alternative splicing anomalies in the presynaptic granule neuron, and by CaMKIIα-driven dendritic spine collapse in the postsynaptic Purkinje neuron. 

### 2.8. Molecular Assessment of Glutamatergic Synapse Strength and Adhesion

**Figure 7:**
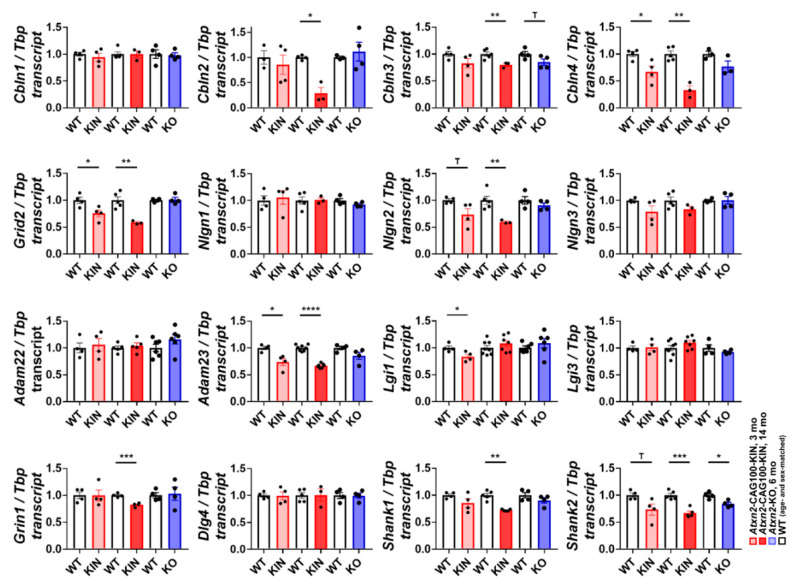
Expression analyses of synaptic structure, transmission and dendrite morphology factors. In *Atxn2*-CAG100-KIN cerebellum throughout disease course at 3 mo and 14 mo of age and in *Atxn2*-KO cerebellum, transcript levels of Cerebellin isoforms (*Cbln1-4*), ionotrophic glutamate receptor δ2 (*Grid2*) and Neuroligin isoforms (*Nlgn1-3*) were examined as extracellular and postsynaptic interactors of Neurexins in maintaining synaptic integrity. The structural bridge of glutamatergic synapses consisting of *Adam22*, *Adam23*, *Lgi1* and *Lgi3*, together with ionotrophic glutamate receptor NMDA type 1 (*Grin1*) involved in synaptic transmission, post-synaptic density markers PSD95 (*Dlg4*) and Shank isoforms (*Shank1-2*) were also quantified throughout disease course by RT-qPCR. Student’s *t*-test with Welch’s correction; 0.05 < *p* < 0.1 ^T^, *p* < 0.05 *, *p* < 0.01 **, *p* < 0.001 ***, *p* < 0.0001 ****. Further information regarding individual fold changes and *p*-values can be found in Supplementary Table S3.

**Figure 8:**
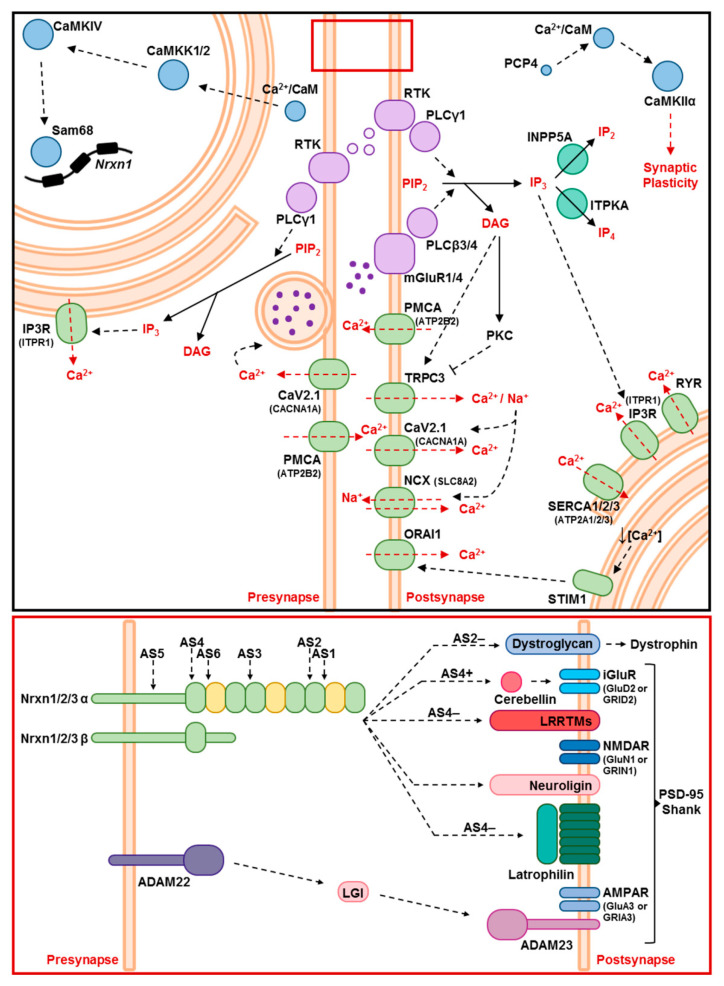
Schematic representation of the Ca^2+^ associated signaling cascades and synaptic integrity components investigated in the framework of this project. Bottom panel corresponds to red inset in the top panel. Neurexin structure and preferential interactions were adapted from Südhof T.C. 2017 [68].

The morphological atrophy of Purkinje dendrite spines and the altered Neurexin-1 splicing suggest impaired synaptic strength and adhesion. Parallel fiber activity via glutamatergic excitation determines the growth of Purkinje spine postsynaptic compartments, the facilitation of synaptic plasticity, and the interaction stability between pre- and post-synaptic structures. Differentially spliced isoforms of *Nrxn1* were previously shown to interact with postsynaptic ionotrophic Glutamate receptor δ2 (*Grid2* encoding GluD2) via extracellular cerebellins (*Cbln1-4*), neuroligins (*Nlgn1-3*), dystroglycan, LRRTMs and latrophilin, to regulate the long-term potentiation and depression features of the synapse [68]. Systematic analyses of cerebellin isoforms revealed an early dysregulation of *Cbln4*, with progressive decrease throughout the disease course, and late-onset downregulations of *Cbln2* and *Cbln3* (Figure 7). Transcript levels for the postsynaptic effector of NRXN1 and Cerebellins, *Grid2*, also showed a significant downregulation, which started at the pre-onset phase and further decreased with disease progression (Figure 7). Another group of postsynaptic NRXN1 interactors, Neuroligins, only showed a significant dysregulation of the *Nlgn2* isoform in parallel with the disease progression (Figure 7). A glutamatergic trans-synaptic adhesion protein complex, which consists of membrane-associated *Adam22*, extracellular *Lgi1* and *Lgi3*, and membrane-associated *Adam23* on the opposite side of the synaptic cleft, serves also as a clustering platform for other synaptic entities such as K^+^ channels or AMPA receptors [76]. Expression analyses of these complex components revealed an early dysregulation of *Adam23* and *Lgi1* at 3 mo, with a progressive decrease of *Adam23* at 14 mo. Interestingly, *Lgi1* showed normal expression at the terminal disease phase in *Atxn2*-CAG100-KIN cerebellum (Figure 7). Finally, transcript levels of ionotropic glutamate receptor NMDA type 1 (*Grin1*) and of the IP3R-associated postsynaptic structural component Shank (*Shank1*, *Shank2*) showed a significant dysregulation at 14 mo. This is in line with the previous observation of dendritic spine pathology in Purkinje cells at 9 mo. *Shank2* also showed an earlier trend towards downregulation at three mo, and a milder dysregulation in *Atxn2*-KO cerebellum. The postsynaptic receptor scaffold PSD95 (*Dlg4*) did not show altered expression throughout the disease course (Figure 7), in good agreement with a previous report that PSD95 binding and relocalization are under the control of CamKII [77]. Together, the data highlight extracellular intermediates *Cbln2* and *Cbln4*, and post-synaptic *Grid2*, *Nlgn2, Adam23* and *Shank2* transcripts as important targets of ATXN2 aggregation pathology, among many other structural synapse components. A schematic representation of all the signaling cascades and transcripts/proteins studied in the context of this work is depicted in Figure 8.

### 2.9. Dissecting the Molecular Signature of SCA2 Pathology to Define Purkinje Neuron Contribution

In order to clarify which dysregulated molecules in the *Atxn2*-CAG100-KIN cerebellum at pre-onset and terminal disease stages are due to Purkinje pathology rather than granule neuron affection, we evaluated their cerebellar in situ hybridization data from the public Allen Mouse Brain Atlas. Prominent expression in Purkinje neurons was observed for most calcium homeostasis channels/transporter (see compilation in Supplementary Figure S3), the glutamate receptors *Grm1* and *Grid2*, *Plcb4*, *Camk2a*, *Khdrbs1*, as well as the adhesion factors *Adam23* and *Nlgn2-3* (Supplementary Figures S4 and S5). Prominent expression in granule neurons was detected for *Plcg1*, *Camkk2*, *Camk4*, the adhesion factors *Cbln1-4* and *Nlgn1* (Supplementary Figures S4 and S5). 

It is relevant to understand whether the postsynaptic pathology is secondary to abnormal presynaptic input, or cell-autonomous. To address this question, we examined several prominent changes that progressed throughout the disease, using another mouse model of SCA2 with human *ATXN2*-Q58 transgenic overexpression under control of the Purkinje cell specific *Pcp2* promoter [78], shortly named Q58-Tg here. Two stages of disease in the Q58-Tg model were investigated, using 16-week (wk) and 46-wk-old cerebella, in accordance with the behavioral and neuropathology data reported earlier [78]. Among the investigated Ca^2+^ transporters, only *Itpr1* showed a rather noteworthy downregulation at early disease stage, with no alteration at late stage. *Ryr1*, *Inpp5a*, *Camk2a*, *Camk4*, *Khdrbs1*, *Nrxn1*, *Nrxn2*, *Nrxn3*, *Nrxn1* AS4 splicing, *Grid2* or *Adam23* were not found dysregulated at any point in disease duration (Supplementary Figure S6). The only other significant finding was the upregulation of the postsynaptic adhesion factor *Nlgn2* at younger age in Q58-Tg cerebellum, which again was not observed in old cerebella. Thus, almost all of the many dysregulations observed in the *Atxn2*-CAG100-KIN mouse cerebellum appear to occur in granule neurons or to occur in Purkinje neurons due to altered glutamatergic input from granule neurons. Only two of the dysregulations could be attributed to Purkinje neuron events in a cell-autonomous manner. 

## 3. Discussion

Generation of the *Atxn2*-CAG100-KIN mouse allowed us to employ cerebellum from this authentic SCA2 model, to define molecular markers of pathology at the pre-onset stage and the terminal stage. The first survey of unbiased global transcriptome profiles and its systematic targeted validation defined a physiological role of ATXN2 for calcium homeostasis and calcium-dependent signaling, while the neurotoxic progressive aggregation of ATXN2 has a stronger impact and affects signaling cascades more broadly, including synaptic strength via glutamate receptors, inositol second messengers, stimulus-dependent allelic splicing, and adhesion factors. The downregulation of about 50 factors in these pathways was assessed regarding progression across lifespan, determining the impact of gain-of-toxic-function versus loss-of-physiological-function of ATXN2 in each case. Prominent disease-associated mRNA markers with significant early downregulation and strongly progressive deficits included *Atp2a2*, *Trpc3*, *Gria3*, *Inpp5a*, *Itpka*, *Camkk2*, *Camk2a*, *Camk2b*, *Camk4*, *Cbln4*, *Grid2* and *Adam23*. Overall, the emerging scenario indicates that the marked Purkinje neuron degeneration to occur largely within the framework of impaired connectivity and stimuli from the glutamatergic granule neurons in the cerebellum. 

*Itpr1* mRNA downregulation was significant, but not as strong as expected from the Q58-Tg mouse model, in view of the reported abnormal interaction between expanded ATXN2 and the ITPR1 protein as a potential cause of pathology in Purkinje neurons [79]. Even in the Q58-Tg mouse, the *Itpr1* downregulation was not progressive with age. Of course, the disease mechanisms may result from a combination of pathways and protein interactions, the role of key molecules is not mutually exclusive, and additional abnormal interactions of ATXN2 beyond ITPR1 may contribute. Interestingly, two of the other progression markers have also been implicated among the primary causal events in the pathogenesis of SCA2. 

Firstly, Sam68, as a K-homology domain-containing RNA-binding signal transduction factor, which is under the control of CaMKIV and of the insulin receptor, was found to co-immunopurify with Ataxin-2 in several species [80]. It was therefore noteworthy that Sam68 protein was elevated in a tissue where ATXN2 aggregates accumulate progressively, and might reflect sequestration into the cytosolic inclusion bodies. Although our immunohistochemical studies observed a granular nuclear redistribution instead, it cannot be excluded that Sam68 is also relocalized to cytosolic stress granules by the expanded ATXN2, where its epitopes may be masked from detection. The sequestration of Sam68 into intranuclear RBP aggregates was shown to be an early disease event and crucial in fragile-X-tremor-ataxia-syndrome (FXTAS), impacting the mRNA splicing/metabolism in dendrites and thus determining the number of glutamatergic synapses [81,82].

Secondly, the *Camk2a* mRNA may have a crucial role in initial pathogenesis, given that *Drosophila melanogaster* studies showed Ataxin-2 to modulate olfactory habituation via CaMKIIα, and demonstrated the *Camk2a* mRNA to copurify with Ataxin-2 in fly head extracts, so it might be a direct mRNA target of Ataxin-2 binding and regulation effects [83]. The impact of fly Ataxin-2 as well as mouse Ataxin-2 on CaMKIIα is phylogenetically conserved, and it may explain the selective atrophy of nervous tissue better than the conserved effect of Ataxin-2 on mTORC1. CaMKIIα is a central hub for the regulation of the electrophysiological long-term potentiation in glutamatergic synapses, for the subsequent biochemical and morphological adaptations of synaptic strength known as plasticity, and for functional consequences such as motor learning [84,85,86,87]. Its progressive deficiency in the *Atxn2*-CAG100-KIN cerebellum is expected to cause a visible atrophy of the dendritic spines in Purkinje neurons, a process that became conspicuous and statistically significant at the age of 9 mo. In the global transcriptome profile of 3-month-old *Atxn2*-CAG100-KIN cerebellum, the strongly significant and massive downregulation of several growth-associated factors such as *Igfbp5* and *Prkcd* may represent very early events in this process of synaptic atrophy. 

Alternative splicing is a known downstream effect of several known ATXN2 protein interactors, with a contributing role to the neurodegenerative process, such as TDP-43, RBFOX1, and Sam68. In the disease process of SCA2, it is unclear at present what the crucial mRNA targets of such splice anomalies can be. Neurexin-1 (*Nrxn1*) splicing is under the control of Sam68, its role as presynaptic receptor for the Neuroligins (*Nlgn1-3)* and the Cerebellins (*Cbln1-4)*, with subsequent effects on postsynaptic GluD2 and Adam23 [88,89], may be responsible for the downregulation of these factors, and all these anomalies together suggest that synaptic adhesion is impaired. Of course, the *Camk2a* mRNA itself can also be alternatively spliced, and numerous other excitability factors in neural tissue are regulated in dependence on trophic stimuli versus stress, via alternative splicing or by alternative polyadenylation, so extensive studies at genome-wide levels will be necessary to obtain a systematic overview.

A question that remains unclear is the role of calcium homeostasis in this process—is its disruption only a consequence of the altered RNA processing and protein interactions in SCA2, or does it have a causal role promoting the aggregation of expanded ATXN2 protein? Several lines of evidence indicate that ATXN2 expansion affects one of the most important neuronal processes, namely the regulation of calcium (Ca^2+^) flux and homeostasis, probably at the ER and at mitochondria. Studies in a transgenic mouse model with overexpression of Q58-expanded human *ATXN2* in cerebellar Purkinje neurons showed that mutant ATXN2 physically interacts with inositol-1,4,5-trisphosphate (IP_3_) receptor (IP3R), which was not observed in healthy animals. Mice with transgenic overexpression of Q127-expanded ATXN2 show decreased firing rates of Purkinje neurons that precede motor deficits [90]. Direct interaction of ATXN2 was proposed to increase IP3R sensitivity to activation by IP_3_, and leads to an enhanced cytosolic Ca^2+^ burst upon glutamatergic stimulation in primary Purkinje cell culture [79]. Overexpression of the IP_3_ phosphatase INPP5A, thus reducing IP3R activation in mutant Purkinje cells, was reported to decrease Purkinje cell death, regulate firing patterns and alleviate motor incoordination [91]. In addition to several other transgenic overexpression models, an *Atxn2*-CAG42 knock-in (KIN) mouse model was generated, which showed very mild neurological disease signs towards the end of normal mouse lifespan without obvious metabolic alterations [22]. Investigation of *Atxn2*-CAG42-KIN mouse cerebellum and its comparison to *Atxn2*-KO mice revealed similar downregulations of Ca^2+^ homeostasis pathway components such as *Itpr1*, *Atp2a2* and *Inpp5a* [59]. Moreover, *Atxn2*-CAG42-KIN cerebella showed increased IP3R levels in the insoluble protein fraction, suggesting its accumulation in the aggregates. However, co-immunoprecipitation experiments did not show a direct interaction of IP3R with expanded ATXN2, contrasting with previous observations in overexpression mutants [59,79]. Machado–Joseph disease (also known as Spinocerebellar ataxia type 3) is caused by polyQ expansions of Ataxin-3, and it was shown that the mutant disease protein does not undergo aggregation in fibroblasts, induced pluripotent stem cells, glia cells, but only in neurons after stimulation by glutamate in processes that depended on Ca^2+^ [92]. In the case of SCA2 pathogenesis, deficient or abnormal stimulation of Purkinje neurons by parallel fibers and erroneous retrograde feedback within the circuit might lead to non-physiological release and reuptake of Ca^2+^ within synapses and neuronal soma, thus promoting the aggregation tendency of expanded ATXN2. This notion is supported by our observation that the Ca^2+^ modulator thapsigargin, but not the glycosylation modulator tunicamycin enhance the interaction of RNA-binding proteins such as ATXN2 and PABP within granular cytosolic structures, as an unspecific effect on proteins and RNAs that is not blocked by the genetic ablation of Ataxin-2. Although the thapsigargin treatment also caused ER stress features in cultured cells, in brain tissue, the ATXN2 aggregation is clearly progressive, while the ER stress at terminal stages of disease is not prominent. Overall, the availability of drugs that enhance or inhibit glutamate stimulation, inositol signals and calcium homeostasis, combined with the generation of authentic SCA2 animal models, will help us to distinguish primary events of SCA2 pathogenesis from the unspecific downstream affection of cerebellar circuit efficiency. 

A final consideration should focus on the physiological function of ATXN2 for these signaling pathways. Given that the genetic ablation or knockdown of Ataxin-2 via the suppression of its physiological roles is able to mitigate and postpone the neurodegenerative process in several disorders (spinocerebellar ataxia type 1, known as SCA1, fronto-temporal lobar degeneration/dementia known as FTLD, amyotrophic lateral sclerosis known as ALS, tauopathies) [55,93,94], it is critical to ask which of the above factors are also modulated in the *Atxn2*-KO mouse cerebellum. Among the promising markers of disease progression summarized in the first Discussion paragraph, only *Trpc3*, *Inpp5a*, *Itpka*, *Camk2a* and *Camk4* show significant impact by the ATXN2 deficiency, and may therefore be core elements of the neurodegenerative process, while the glutamate receptors and the adhesion factors might represent secondary players. Interestingly, the downregulated expression of these five factors in the *Atxn2*-CAG100-KIN cerebellum at pre-onset stage is on average as strong as in the *Atxn2*-KO cerebellum, but at terminal stage, about one third stronger than the *Atxn2*-KO. This observation suggests that a cerebellar phenotype does not appear in the *Atxn2*-KO because the deficit is non-progressive and compensated by other molecules within the same pathway, while the *Atxn2*-CAG100-KIN develops a cerebellar phenotype when the aggregation process sequestrates ATXN2 interactor molecules within the relevant pathway into insolubility, progressively eliminating the compensatory players and challenging multiple stress response pathways (i.e., ERAD, autophagy, mitochondrial dysfunction, energy deprivation) to be activated simultaneously. These mouse data on the role of Ataxin-2 in signal transduction and integration are compatible with the previous fly observations that Ataxin-2 modulates the habituation of sensory input in the olfactory system [83,95]. Sensory habitation was shown to depend also on other RNA-binding proteins, such as FMR1, both in flies and rodents [96,97]. 

Jointly, the understanding of molecular expression profiles at different ages of *Atxn2*-CAG100-KIN cerebellum and its comparison to that of *Atxn2*-KO is essential to elucidate the molecular chain of causality in the onset and progression of the pathology. The precise identification of native ATXN2 target pathways vs. expansion/aggregation effects is crucial. This approach identifies useful molecular markers to assess the benefit of neuroprotective treatment approaches, at a time when the utilization of *ATXN2*-ASOs for the treatment of SCA2 and ALS in clinical trials is imminent. The data presented here will also help to understand potential side effects of an ATXN2 knockdown approach.

## 4. Materials and Methods 

### 4.1. Animals and Genotyping

The generation, housing and genotyping of both *Atxn2*-CAG100-knock in (KIN) and *Atxn2*-knock out (KO) lines were described before [23,48]. The generation of *ATXN2*-Q58-Tg line was described earlier [78], and the genotyping was done according to the published protocol. All animals were housed in individually ventilated cages with 12 h light/dark cycle at the Central Animal Facility (ZFE) of the Goethe University Medical School in Frankfurt am Main, Germany. All animal experiments were performed in accordance with the German Animal Welfare Act, the Council Directive of 24th November 1986 (86/609/EWG) with Annex II, and the ETS123 (European Convention for the Protection of Vertebrate Animals), and were reviewed by the Regierungspräsidium Darmstadt with approval code V54-19c20/15-FK/1083 (approved on 27th of March, 2017).

### 4.2. Transcriptome Screening

Single-stranded cDNA library was generated from DNase treated 1 μg of total RNA (3 WT vs. 3 *Atxn2*-CAG100-KIN cerebella), using GeneChipTM WT PLUS Reagent Kit (Applied Biosystems, Thermo Scientific, Waltham, MA, USA). Fragmentation and labeling of the cDNA library was performed immediately before hybridization to a Clariom D Array (Thermo Scientific, Waltham, MA, USA). The microarrays were scanned with the Affymetrix GeneChip Scanner, and data analysis was done with the Transcriptome Analysis Console (TAC) 4.0.1 (Applied Biosystems, Thermo Scientific, Waltham, MA, USA) using default parameters. For STRING interaction and pathway enrichment analysis, default parameters were employed.

### 4.3. Cell Culture and Treatments

Different clones of WT and *Atxn2*-KO murine embryonal fibroblasts (MEFs) were generated and cultured as described before [34], in growth medium consisting of DMEM (4.5 g/L glucose, Gibco, Thermo Scientific, Waltham, MA, USA), 10% FCS (Gibco, Thermo Scientific, Waltham, MA, USA), 2 mM L-Glutamine (Gibco, Thermo Scientific, Waltham, MA, USA) and 1% Penicillin-Streptomycin (Gibco, Thermo Scientific, Waltham, MA, USA). ER stress inducers Thapsigargin (TG) (Sigma Aldrich, St. Louis, MO, USA) and Tunicamycin (TM) (Sigma Aldrich, St. Louis, MO, USA) were added in the normal growth medium in various final concentrations and time intervals, as indicated on the figures.

### 4.4. RNA Isolation and Expression Analyses

Cerebellar tissue from WT and homozygous *Atxn2*-CAG100-KIN and *Atxn2*-KO animals were dissected and immediately frozen in liquid nitrogen. The numbers of animals used in transcript expression analyses are as follows: 4 WT vs. 4 *Atxn2*-CAG100-KIN at 3 mo, 5 WT vs. 3 *Atxn2*-CAG100-KIN at 14 mo, and 4 WT vs. 4 *Atxn2*-KO at 6 mo of age. RNA extraction from frozen tissue samples and MEFs was performed with the TRIzol Reagent (Sigma Aldrich, St. Louis, MO, USA) following manufacturer’s instructions. cDNA synthesis was done from 1 µg of total RNA using the SuperScript IV VILO kit (Thermo Scientific, Waltham, MA, USA), according to the user’s manual. Transcript expression analyses were done by quantitative real-time PCR utilizing StepOnePlus™ Real-Time PCR System (Applied Biosystems, Thermo Scientific, Waltham, MA, USA). For quantification with TaqMan^®^ Assays, each qPCR reaction consisted of cDNA from 25 ng total RNA, 1 µL TaqMan^®^ Assay (Applied Biosystems, Thermo Scientific, Waltham, MA, USA), 10 µL FastStart Universal Probe Master 2x (Rox) Mix (Roche, Basel, Switzerland) and ddH2O up to 20 µL of total volume. All TaqMan^®^ Assays utilized in this study are listed in Table 1. Each sample was measured in duplicate with the following cycling conditions: 50 °C for 2 min, 95 °C for 10 min, followed by 40 cycles of 95 °C for 15 s and 60 °C for 1 min. Expression data were analyzed using 2^−ΔΔCt^ method [98] with *Tbp* as housekeeping gene.

For quantification with SYBR^®^ Green primers (Merck, Darmstadt, Germany), each qPCR reaction consisted of cDNA from 25 ng total RNA, 5 pmol/μL primers, 10 µL qPCR Mastermix Plus for SYBR Green I (Eurogentec, Liège, Belgium) and ddH_2_O up to 20 µL of total volume. All SYBR^®^ Green primers utilized in this study are listed in Table 2. Each sample was measured in duplicates with the following cycling conditions: 95 °C for 10 min, followed by 40 cycles of 95 °C for 15 s and 60 °C for 1 min, and a melt curve stage of 95 °C for 15 s, 60 °C for 1 min and 95 °C for 15 s. Expression data were analyzed using 2^−ΔΔCt^ method [98] with *Actb* as housekeeping gene.

### 4.5. Protein Extraction and Quantitative Immunoblots

Frozen cerebella from 5 WT vs. 5 homozygous *Atxn2*-CAG100-KIN animals were homogenized with a motor pestle in 5–10x weight/volume amount of RIPA lysis buffer (50 mM Tris-HCl pH8.0, 150 mM NaCl, 1% NP-40, 0.5% sodium deoxycholate, 0.1% SDS, 2 mM EDTA, and Protease Inhibitor Cocktail, cOmplete™, Mini, EDTA-free (Roche, Basel, Switzerland)). Following centrifugation at 13,000× *g* for 15 min at 4 °C, supernatant was transferred to a fresh tube, and the pellet was homogenized in 2x SDS lysis buffer (137 mM Tris-HCl, pH 6.8, 4% SDS, 20% glycerol) with a motor pestle, after which, samples were sonicated with an ultrasonic homogenizer (Bandelin, Berlin, Germany). Aliquots with 20 µg of total protein from the RIPA lysates and 10 µg of total protein from the SDS lysates were mixed with 2x Loading Buffer (250mM Tris-HCl pH 7.4, 20% Glycerol, 4% SDS, 10% 2-Mercaptoethanol, 0.005% Bromophenol blue, 5% ddH2O) and incubated at 90 °C for 3 min. Proteins were separated on polyacrylamide gels and transferred to Nitrocellulose membranes (Protran, GE Healthcare, Chicago, IL, USA). The membranes were blocked in 5% BSA/TBS-T for 1 h, and incubated overnight at 4 °C, with primary antibodies diluted in blocking buffer. The primary antibodies utilized in this study are: ACTB (Sigma Aldrich, St. Louis, MO, USA, A5441, 1:10,000), ATP2A2 (Cell Signaling, Danvers, MA, USA, #9580, 1:1000), CaMKIIα (Cell Signaling, Danvers, MA, USA, #3357, 1:1000), CaMKIV (Cell Signaling, Danvers, MA, USA, #4032, 1:500), GluA3 (Cell Signaling, Danvers, MA, USA, #4676, 1:1000), ITPR1 (Abcam, Cambridge, UK, ab5804, 1:500), and Sam68 (Abclonal, Woburn, MA, USA, A6101, 1:1000). Membranes were washed and incubated in fluorescent-labeled secondary antibodies diluted in blocking buffer for 1 h at RT. Secondary antibodies used in this study are: goat anti-mouse (Licor Biosciences, Lincoln, NE, USA, 926-32280 IRDye 800CW or 926-68070 IRDye 680RD, 1:10,000) and goat anti-rabbit (Licor Biosciences, Lincoln, NE, USA, 926-32211 IRDye 800CW or 926-68071 IRDye 680RD, 1:10,000). Membranes were scanned using Li-Cor Odyssey Classic instrument (Licor Biosciences, Lincoln, NE, USA), and image analyses were performed on ImageStudio software.

### 4.6. Colorimetric Ca^2+^ Measurement

Colorimetric Calcium Detection Assay Kit (Abcam, Cambridge, UK) was utilized for the measurement of total Ca^2+^ concentrations in cytosolic and membrane-encapsulated organelle-rich fractions of mouse cerebellum following manufacturer’s protocol with minor modifications in the sample lysis step. Fresh cerebellar tissue from 7 WT vs. 6 homozygous *Atxn2*-CAG100-KIN animals at the age of 3 mo were homogenized with a motor pestle in 5-10x weight/volume amount of low detergent PN Buffer (1x PBS, 1% NP-40, 150 mM NaCl) immediately after dissection. Following centrifugation at 13,000× *g* for 15 min at 4 °C, supernatant was transferred to a fresh tube, and the pellet was homogenized in high-detergent Urea Lysis Buffer (8 M Urea, 10 mM Tris(2-carboxyethyl)phosphine, 40 mM 2-Chloroacetamide, 100 mM Tris) with a motor pestle, after which samples were sonicated with an ultrasonic homogenizer (Bandelin, Berlin, Germany). Colorimetric Calcium Detection Assay Kit was used according to user manual from this point on, each sample being measured in duplicates. The Ca^2+^ standard provided in the kit was used to generate a standard curve and determine the Ca^2+^ concentrations of unknown samples. Densitometric measurements were performed with Spark^®^ multimode microplate reader (Tecan Technologies, Zürich, Switzerland) by measuring the absorbance value at 575 nm. Final concentration calculations were done in Microsoft Excel software following the instructions of the manufacturer.

### 4.7. Immunostainings

For immunocytochemistry, 5 × 10^4^ cells from WT and *Atxn2*-KO MEF cultures were seeded on 12 mm cover slips. The next day, the cells were stressed with 5 µM TG and 10 µg/mL TM supplemented in the growth medium for 6 h at 37 °C, then were fixed with 4% PFA/PBS at RT for 20 min. Following permeabilization with 0.1% Triton-X-100/PBS for 20 min at RT, and blocking with 3% BSA/PBS solution for 1 h at RT, cells were incubated in primary antibody solution for 1 h at RT with anti-ATXN2 (BD Biosciences, Franklin Lakes, NJ, USA, #611378, 1:100) and PABP (Abcam, Cambridge, UK, ab21060, 1:250), diluted in blocking buffer. After 3× washing in PBS, secondary antibody incubation with chicken anti-mouse-Alexa Fluor 488 (Molecular Probes, Eugene, OR, USA, A21200, 1:1000), goat anti-rabbit-Alexa Fluor 546 (Molecular Probes, Eugene, OR, USA, A11036, 1:1000) and DAPI (Thermo Scientific, Waltham, MA, USA, 1 µg/mL) diluted in blocking buffer was performed for 1 h at RT in dark. Coverslips were mounted with Lab Vision™ PermaFluor™ fluorescent mounting medium (Thermo Scientific, Waltham, MA, USA) on glass slides and dried overnight. Imaging was done with Zeiss Axiovert 200M (Carl Zeiss, Oberkochen, Germany) inverted microscope using a 100× objective, and image processing was done with ImageJ software.

For immunohistochemistry, 14-month-old WT and homozygous *Atxn2*-CAG100-KIN mice were deeply anesthetized with intraperitoneal Ketaset (300 mg/kg) and Domitor (3 mg/kg). Intracardial perfusion was performed with PBS for 5 min and 4% PFA in 0.1 M PBS for 5 min. Then, the tissue was post-fixed overnight in 4% PFA at 4 °C, immersed in 30% sucrose until it sank to the bottom, cryosectioned to 30 μm sagittal slices and stored at −20 °C in cryoprotection buffer (30% ethylene glycol, 25% glycerin, 0.01% sodium azide in 0.1 M PBS). Free floating cryosections were washed 3× for 10 min with 0.3% Triton-X-100/PBS and blocked with 5% goat serum (Sigma Aldrich, St. Louis, MO, USA) in 0.1% Triton-X-100/PBS for 1 h at RT with slow shaking. Primary antibody solution with anti-ATXN2 (BD Biosciences, Franklin Lakes, NJ, USA, #611378, 1:100) and anti-SAM68 (Abclonal, Woburn, MA, USA, A6101, 1:1000) diluted in blocking buffer was performed at 4 °C with slow shaking overnight. Sections were washed 3 × 10 min with PBS and incubated in secondary antibody solution for 2 h at RT in dark with goat anti-mouse-Alexa Fluor 546 (Molecular Probes, Eugene, OR, USA, A11003, 1:1000), goat anti-rabbit-Alexa Fluor 488 (Molecular Probes, Eugene, OR, USA, A11034, 1:1000) and DAPI (Thermo Scientific, Waltham, MA, USA, 1 µg/mL) diluted in blocking buffer. After 3 × 10 min washes in PBS, sections were mounted on glass slides with Lab Vision™ PermaFluor™ fluorescent mounting medium (Thermo Scientific, Waltham, MA, USA) and dried overnight. Imaging was done with Nikon Eclipse TE2000-E (Nikon, Tokyo, Japan) inverted confocal microscope using a 40× objective, and image processing was done with Fiji BioVoxxel software.

### 4.8. Silver Impregnation

Silver impregnation was done as described before [100]. Since Purkinje cell morphology differs between lobes, only cells from lobules 1 to 6 have been evaluated. In addition, only cells were selected for analysis, which were stained throughout most of the cell and which were positioned isolated from other stained cells so that the whole dendritic profile was visible. Images were taken with a confocal microscope (TCS SP2, Leica, Wetzlar, Germany), having a 40× oil immersion objective (NA = 1.25) using a zoom factor of 1.8 (lower magnifications) and 6.0 (higher magnifications). Voxel sizes have been set to 0.2 µm (x and y) and 2.0 µm (z) for lower magnifications, and 0.06 (x and y) and 0.5 (z) for higher magnifications. Z stacks were imported into ImageJ (v 1.53c) and an extended depth of field calculation was done using ImageJ Plugin of Forster et al. [101]. Three animals have been prepared from each genotype, at least 10 sections from the vermal region have been stained and 1–2 Purkinje cells of each slice have been selected and photographed. For each Purkinje cell 3–4 dendritic regions have been selected and spines were measured along a segment which was defined by the following criteria: (i) it is part of the terminal endings, (ii) it is visible along a length of at least 5 µm, and (iii) the segment was visible within 10 z planes. Using the segmented line tool in ImageJ, the lengths of spines were measured and combined for each Purkinje cell. In order to validate measurement, images of unknown genotype to the investigator were analyzed by three different experimentators (NC, SLB and NES) and results were compared. There was a 95% confidence of obtaining the same results. For statistical evaluation, equal data distribution was assessed with Levene’s test, and one-way ANOVA was used to compare genotype dependent spine number and length differences.

### 4.9. Statistical Analyses

Unless specified otherwise, all statistical tests for comparisons between WT and mutant mice (except for silver impregnation analyses) were performed using unpaired Student’s *t*-test with Welch’s correction, and for cell culture experiments using 2-way ANOVA with multiple testing corrections on GraphPad Prism software version 7. The fold-change differences and *p*-values of all the expression analyses performed in mouse tissue and cell culture are listed in Supplementary Table S3. Graphs display mean values with standard error of the mean (s.e.m.). Values *p* < 0.05 were considered significant and marked with asterisks *p* < 0.05 *, *p* < 0.01 **, *p* < 0.001 ***, *p* < 0.0001 ****. T indicates a trend towards statistical significance (0.05 < *p* < 0.1).

## 5. Conclusions

The *Atxn2*-CAG100-KIN mouse holds the advantage of modelling ATXN2-dependent neurological disease, not only in a targeted neuron population, but also in the whole organism. Studying different stages of disease progression in this SCA2 model, and especially the primary pathogenesis events at pre-onset phase, is crucial to dissect the causal chains of molecular dysregulations as the crucial effectors of pathology, and to identify potential targets for preventive therapeutic interventions. The global transcriptome profile of *Atxn2*-CAG100-KIN cerebellum at pre-onset phase highlights Ca^2+^ homeostasis and associated downstream effectors, such as CaMK signaling and glutamatergic neurotransmission, as the prominent targets of early-stage pathology. Progressively altered expression levels of various Ca^2+^ channels and transporters indicate an imbalanced Ca^2+^ localization between cytosol and ER. Normally diffuse cytosolic ATXN2 protein was found to relocalize into stress granules upon thapsigargin-triggered ER stress via enforced Ca^2+^ imbalance, but not upon ER stress via blocking glycosylation. In accordance with their dependence on Ca^2+^ homeostasis, CaMKIIα and CaMKIV signaling pathways and their molecular outcomes, i.e., post-synaptic dendritic spine morphology in Purkinje neurons and pre-synaptic alternative splicing of Neurexins in granule neurons, were found to be affected by ATXN2 pathology. These initial findings were further supported by subsequent dysregulations of numerous pre- and post-synaptic adhesion factors at the granule cell-Purkinje neuron interface, suggesting a simultaneous onset and progression of pathology in different neuron types in the cerebellum.

## Figures and Tables

**Table 1:** List of TaqMan^®^ Assays utilized in this study.

Transcript	Assay ID	Transcript	Assay ID	Transcript	Assay ID
*Adam22*	Mm01316488_m1	*Ern1(Ire1)*	Mm00470233_m1	*Nrxn1*	Mm03011136_m1
*Adam23*	Mm00478606_m1	*Gria3*	Mm00497506_m1	*Nrxn2*	Mm01236851_m1
*Atf4*	Mm00515324_m1	*Grid2*	Mm00515053_m1	*Nrxn3*	Mm00553213_m1
*Atf6*	Mm01295319_m1	*Grm1*	Mm00810219_m1	*Orai1*	Mm00774349_m1
*Atp2a1*	Mm01275320_m1	*Grm4*	Mm01306128_m1	*Pcp4*	Mm00500973_m1
*Atp2a2*	Mm01201431_m1	*Hspa5(Bip)*	Mm00517691_m1	*Plcb3*	Mm00476953_m1
*Atp2a3*	Mm00443898_m1	*Hspa9(Grp75)*	Mm00477716_g1	*Plcb4*	Mm00649825_m1
*Atp2b2*	Mm00437640_m1	*Inpp5a*	Mm00805812_m1	*Plcg1*	Mm01247293_m1
*Atxn2*	Mm01199894_m1	*Itpka*	Mm00525139_m1	*Prkcd*	Mm00440891_m1
*Cacna1a*	Mm00432190_m1	*Itpr1*	Mm00439907_m1	*Ryr1*	Mm01175211_m1
*Camk2a*	Mm00437967_m1	*Khdrbs1*	Mm00516130_m1	*Ryr3*	Mm01328421_m1
*Camk2b*	Mm00432284_m1	*Khdrbs2*	Mm00504671_m1	*Shank1*	Mm01206737_m1
*Camk2d*	Mm00499266_m1	*Khdrbs3*	Mm00501666_m1	*Shank2*	Mm00683065_m1
*Camk2g*	Mm00618047_m1	*Lgi1*	Mm01198941_m1	*Sigmar1*	Mm00448086_m1
*Camk4*	Mm01135329_m1	*Lgi3*	Mm00507490_m1	*Slc8a2*	Mm00455836_m1
*Camkk1*	Mm00517053_m1	*Mcu*	Mm01168773_m1	*Smdt1*	Mm01306306_m1
*Camkk2*	Mm00520236_m1	*Mfn1*	Mm00612599_m1	*Stim1*	Mm01158413_m1
*Cbln1*	Mm01247194_g1	*Mfn2*	Mm00500120_m1	*Tbp*	Mm00446973_m1
*Cbln2*	Mm01261557_g1	*Micu1*	Mm00522783_m1	*Trpc3*	Mm00444690-m1
*Cbln3*	Mm00490772_g1	*Micu2*	Mm00551312_m1	*Vdac1*	Mm00834272_m1
*Cbln4*	Mm00558663_m1	*Micu3*	Mm01194824_m1	*Xbp1s*	Mm03464496_m1
*Ddit3(Chop)*	Mm00499207_m1	*Nlgn1*	Mm02344307_m1	*Xbp1u*	Mm03464497_s1
*Dlg4(Psd-95)*	Mm00492193_m1	*Nlgn2*	Mm01703404_m1		
*Eif2ak3(Perk)*	Mm00438708_m1	*Nlgn3*	Mm01225951_m1		

**Table 2:** List of mouse-specific SYBR^®^ Green primers utilized in this study.

Primer Name	Sequence (5′ → 3′)	Reference
AS1 − Fwd	ACTGCAGCCAAGGAAAAGAAGAGTA	
AS1 − Rev	GTTTTAAAGGACAGAGTTATTTCAT	
AS2 − Fwd	TCTGCGTCAGGTGACAATATCAG	
AS2 + Fwd	CTCAGGCATTGGACACGCTA	
AS2 −/+ Rev	GAAGGTCGGCTGTGCTGGGG	Nguyen et al. 2016 [99]
AS3 − Fwd	TCAATCTAGGCAAAGGTCCTG	
AS3 + Fwd	TTGTATCAGGATTAACTGTAATTCC	
AS3 −/+ Rev	TTTCCTCGCCGAACCACACG	Iijima et al. 2011 [67]
AS4 − Fwd	CGCTACCCTGCAGGGCGTCAGCTCAC	
AS4 + Fwd	TAGTTGATGAATGGCTACTCGACAAA	Iijima et al. 2011 [67]
AS4 −/+ Rev	GACTCAGTTGTCATAGAGGAAGGCAC	Iijima et al. 2011 [67]
AS5 − Fwd	AGCCAGCCAACCCCACCAGAGTA	
AS5 + Fwd	AGATGACATCCTTGTGGCCT	
AS5 −/+ Rev	ACCATGCCAGTGGTACTGCT	
AS6 − Fwd	ATGCGAAGGGCCCAGCA	
AS6 + Fwd	GCATTGATGAAAGCTGACTTGC	
AS6 −/+ Rev	GGAAGTCATGCTACAGTCACAGC	
*Actb* Fwd	GGAAATCGTGCGTGACATCAAAG	
*Actb* Rev	CATACCCAAGAAGGAAGGCTGG	

## Supplementary Materials

Supplementary materials can be found at https://www.mdpi.com/1422-0067/21/18/6673/s1.

## Abbreviations

°C	Degrees Celcius
µg	Microgram
µm	Micrometer
μL	Microliter
μM	Micromolar
ACTB	Beta Actin
*Adam22*	A Disintegrin And Metalloproteinase Domain 22
*Adam23*	A Disintegrin And Metalloproteinase Domain 23
ALS	Amyotrophic lateral sclerosis
AMPA	α-amino-3-hydroxy-5-methyl-4-isoxazolepropionic acid
ANOVA	Analysis of variance
AS	Alternative splicing
ASO	Antisense oligonucleotide
*Atf4*	Activating Transcription Factor 4
*Atf6*	Activating Transcription Factor 6
*Atp2a1*	ATPase Sarcoplasmic/Endoplasmic Reticulum Ca^2+^ Transporting 1
*Atp2a2*	ATPase Sarcoplasmic/Endoplasmic Reticulum Ca^2+^ Transporting 2
*Atp2a3*	ATPase Sarcoplasmic/Endoplasmic Reticulum Ca^2+^ Transporting 3
*Atp2b2*	ATPase Plasma Membrane Ca^2+^ Transporting 2
ATXN2	Ataxin-2
ATXN2L	Ataxin-2-Like
*BiP*	Endoplasmic Reticulum Lumenal Ca^2+^-Binding Protein Grp78 (*Hspa5*)
BSA	Bovine serum albumin
Ca^2+^	Calcium ion
CAA	Cytosine-Adenine-Adenine trinucleotide
*Cabp7*	Calcium Binding Protein 7
*Cacna1a*	Calcium Voltage-Gated Channel Subunit alpha-1-A
CAG	Cytosine-Adenine-Guanine trinucleotide
CaM	Calmodulin
CaMK	Ca^2+^/Calmodulin-Dependent Protein Kinase
*Camk2a*	Ca^2+^/Calmodulin-Dependent Protein Kinase II alpha
*Camk2b*	Ca^2+^/Calmodulin-Dependent Protein Kinase II beta
*Camk2d*	Ca^2+^/Calmodulin-Dependent Protein Kinase II delta
*Camk2g*	Ca^2+^/Calmodulin-Dependent Protein Kinase II gamma
*Camk4*	Ca^2+^/Calmodulin-Dependent Protein Kinase IV
CaMKIIα	Ca^2+^/Calmodulin-Dependent Protein Kinase II alpha
CaMKIV	Ca^2+^/Calmodulin-Dependent Protein Kinase IV
*Camkk1*	Ca^2+^/Calmodulin-Dependent Protein Kinase Kinase 1
*Camkk2*	Ca^2+^/Calmodulin-Dependent Protein Kinase Kinase 2
*Car8*	Carbonic Anhydrase 8
CaV2.1	Calcium Voltage-Gated Channel Subunit alpha-1-A
*Cbln1*	Cerebellin 1
*Cbln2*	Cerebellin 2
*Cbln3*	Cerebellin 3
*Cbln4*	Cerebellin 4
cDNA	Complementary DNA
*Chop*	C/EBP-Homologous Protein (*Ddit3*)
CNS	Central nervous system
Ct	Cycle threshold
C-terminal	Carboxy-terminal end
DAG	Diacylglycerol
DAPI	4′,6-diamidino-2-phenylindole
ddH2O	Double-distilled water
*Dlg4*	Discs Large MAGUK Scaffold Protein 4
DMEM	Dulbecco’s modified essential medium
DNA	Deoxyribonucleic acid
DNase	Deoxyribonuclease
EDTA	Ethylenediaminetetraacetic acid
eIF	Eukaryotic Translation Initiation Factor
ER	Endoplasmic reticulum
ERAD	Endoplasmic reticulum—associated degradation
FCS	Fetal calf serum
FMR1	Fragile X Mental Retardation 1
FTLD	Fronto-temporal lobar degeneration
FUS	Fused in Sarcoma
Fwd	Forward primer
FXTAS	Fragile-X-tremor-ataxia syndrome
g	Gram
GC	Granule cell
GCL	Granule cell layer
GluA3	Glutamate Ionotropic Receptor AMPA Type Subunit 3
GluD2	Glutamate Ionotropic Receptor Delta Type Subunit 2
GO	Gene Ontology
*Gria3*	Glutamate Ionotropic Receptor AMPA Type Subunit 3
*Grid2*	Glutamate Ionotropic Receptor Delta Type Subunit 2
*Grin1*	Glutamate Ionotropic Receptor NMDA Type Subunit 1
*Grm1*	Glutamate Metabotropic Receptor 1
*Grm4*	Glutamate Metabotropic Receptor 4
*Grp75*	Glucose-Regulated Protein 75 Kda (*Hspa9*)
h	Hour
HCl	Hydrochloric acid
i.e.,	id est
*Icmt*	Isoprenylcysteine Carboxyl Methyltransferase
*Igfbp5*	Insulin Like Growth Factor Binding Protein 5
*Inpp5a*	Inositol Polyphosphate-5-Phosphatase A
IP3	Inositol-1,4,5-trisphosphate
IP3R	Inositol-1,4,5-Trisphosphate Receptor Type 1
*Ire1*	Inositol-Requiring Protein 1 (*Ern1*)
*Itpka*	Inositol-Trisphosphate 3-Kinase A
*Itpr1*	Inositol-1,4,5-Trisphosphate Receptor Type 1
K+	Potassium ion
kD	Kilo Dalton
KEGG	Kyoto Encyclopedia of Genes and Genomes
kg	Kilogram
*Khdrbs1*	KH RNA Binding Domain Containing, Signal Transduction Associated 1
*Khdrbs2*	KH RNA Binding Domain Containing, Signal Transduction Associated 2
*Khdrbs3*	KH RNA Binding Domain Containing, Signal Transduction Associated 3
KIN	Knock in
KO	Knock out
l	Liter
*Lgi1*	Leucine Rich Glioma Inactivated 1
*Lgi3*	Leucine Rich Glioma Inactivated 3
LRRTM	Leucine Rich Repeat Transmembrane Protein
Lsm	Like-Smith antigen protein domain
Lsm-AD	Lsm-associated domain
M	Molar
MAM	Mitochondria-associated membrane
*Mcu*	Mitochondrial Calcium Uniporter
MEF	Mouse embryonal fibroblast
*Mfn1*	Mitofusin 1
*Mfn2*	Mitofusin 2
mg	Milligram
mGluR	Glutamate Metabotropic Receptor
*Micu1*	Mitochondrial Calcium Uptake 1
*Micu2*	Mitochondrial Calcium Uptake 2
*Micu3*	Mitochondrial Calcium Uptake 3
min	Minute
miRNA	Micro RNA
ml	Milliliter
ML	Molecular layer
mm	Millimeter
mM	Millimolar
mo	Month
mRNA	Messenger RNA
mTORC1	Mechanistic Target of Rapamycin Complex 1
n	Number of samples
NA	Numerical Aperture
NaCl	Sodium chloride
NCX	Na^+^/Ca^2+^-Exchange Protein (*Slc8a2*)
ng	Nanogram
*Nlgn1*	Neuroligin 1
*Nlgn2*	Neuroligin 2
*Nlgn3*	Neuroligin 3
nm	Nanometer
NMDA	N-methyl-d-aspartic acid
No	Number
NP-40	Nonidet P-40 (nonyl phenoxypolyethoxylethanol)
*Nrxn1*	Neurexin 1
*Nrxn2*	Neurexin 2
*Nrxn3*	Neurexin 3
N-terminal	Amino (NH_2_)-terminal end
OPA1	Optic Atrophy Protein 1
*Orai1*	ORAI Calcium Release-Activated Calcium Modulator 1
*p*	Probability value
PABP	Poly(A)-Binding Protein
PAM2	PABP-interacting motif 2
PBS	Phosphate buffered saline
PC	Purkinje cell
PCL	Purkinje cell layer
*Pcp2*	Purkinje Cell Protein 2
*Pcp4*	Purkinje Cell Protein 4 (PEP-19)
PCR	Polymerase chain reaction
PD	Parkison’s disease
*Perk*	PRKR-Like Endoplasmic Reticulum Kinase (*Eif2ak3*)
PFA	Paraformaldehyde
PINK1	PTEN Induced Kinase 1
PIP_2_	Phosphatidylinositol-4,5-bisphosphate
PKC	Protein Kinase C
PLC	Phospholipase C
*Plcb3*	Phospholipase C Beta 3
*Plcb4*	Phospholipase C Beta 4
*Plcg1*	Phospholipase C Gamma 1
PMCA	Plasma Membrane Ca^2+^ Pump (*Atp2b2*)
pmol	Picomole
PolyQ	Poly-Glutamine
PRD	Proline-rich domain
*Prkcd*	Protein Kinase C Delta
*Prmt8*	Protein Arginine Methyltransferase 8
PSD95	Postsynaptic Density Protein 95 (*Dlg4*)
Q	Glutamine
RBFOX1	RNA Binding Fox-1 Homolog 1
RBP	RNA-binding protein
Rev	Reverse primer
*Rgs8*	Regulator of G Protein Signaling 8
RIPA	Radioimmunoprecipitation assay
RNA	Ribonucleic acid
RNase	Ribonuclease
*Rora*	Retinoic Acid Receptor-Related Orphan Receptor Alpha
RT	Room temperature
RTK	Receptor Tyrosine Kinase
RT-qPCR	Reverse-Transcriptase quantitative PCR
*Ryr1*	Ryanodine Receptor 1
*Ryr3*	Ryanodine Receptor 3
sec	Second
s.e.m.	Standard error of the mean
Sam68	Src-Associated In Mitosis 68 KDa Protein (*Khdrbs1*)
SCA1	Spinocerebellar ataxia type 1
SCA2	Spinocerebellar ataxia type 2
SDS	Sodium dodecyl sulfate
*Sema7a*	Semaphorin 7A
SERCA1	Sarcoplasmic/Endoplasmic Reticulum Calcium ATPase 1 (*Atp2a1*)
SERCA2	Sarcoplasmic/Endoplasmic Reticulum Calcium ATPase 2 (*Atp2a2*)
SERCA3	Sarcoplasmic/Endoplasmic Reticulum Calcium ATPase 3 (*Atp2a3*)
SG	Stress granule
*Shank1*	SH3 And Multiple Ankyrin Repeat Domains 1
*Shank2*	SH3 And Multiple Ankyrin Repeat Domains 2
*Sigmar1*	Sigma Non-Opioid Intracellular Receptor 1
*Slc8a2*	Solute Carrier Family 8 Member A2
Slm1	Sam68-Like Mammalian Protein 1 (*Khdrbs2*)
Slm2	Sam68-Like Mammalian Protein 2 (*Khdrbs3*)
*Smdt1*	Single-Pass Membrane Protein with Aspartate Rich Tail 1
*Stim1*	Stromal Interaction Molecule 1
STRING	Search Tool for the Retrieval of Interacting Genes/Proteins
T	Trend
*Tbp*	TATA-binding factor of transcription
TBS-T	Tris-buffered saline with Tween20
TCA	Tricarboxylic acid cycle
TDP-43	TAR DNA-Binding Protein 43 (*Tardbp*)
TG	Thapsigargin
Tg	Transgenic (mouse)
TIA-1	T-Cell-Restricted Intracellular Antigen-1
TM	Tunicamycin
tRNA	Transfer RNA
*Trpc3*	Transient Receptor Potential Cation Channel Subfamily C Member 3
UniProt	Universal Protein resource
UPR	Unfolded protein response
*Vdac1*	Voltage Dependent Anion Channel 1
vs.	*versus*
wk	Week
WT	Wild type
*Xbp1s*	X-Box Binding Protein 1 spliced
*Xbp1u*	X-Box Binding Protein 1 unspliced
