# Supplementary figures and images for: Mouse Ataxin-2 Expansion Downregulates CamKII and Other Calcium Signaling Factors, Impairing Granule—Purkinje Neuron Synaptic Strength
Source: Int J Mol Sci. 2020 Sep 12;21(18):6673. doi: 10.3390/ijms21186673 (PMC7555182; doi:10.3390/ijms21186673)

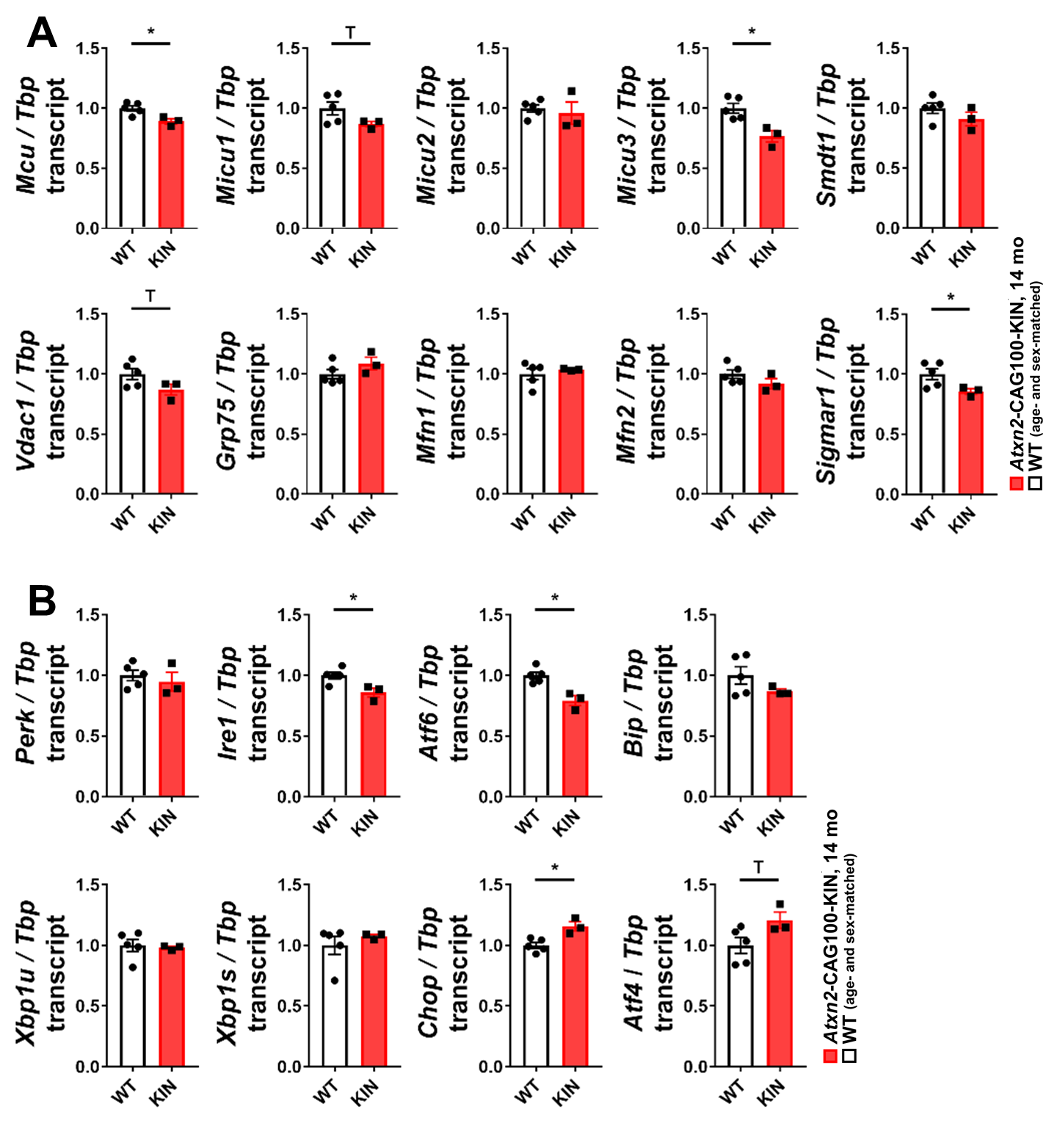

Supplement: Supplementary file 1 [file ijms-21-06673-s001.zip › ArsovicAuburger-SupplFigureS1.TIF]

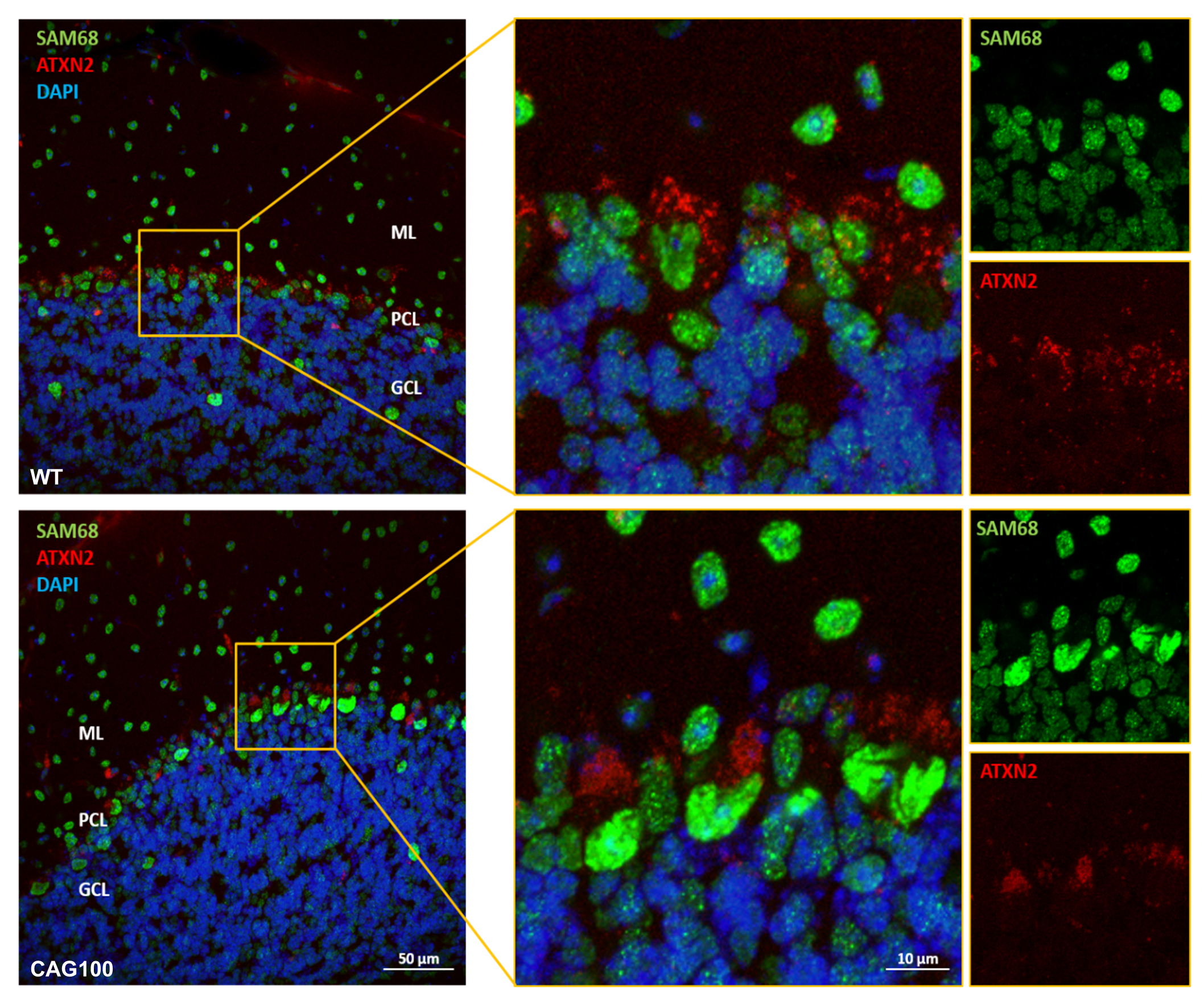

Supplement: Supplementary file 1 [file ijms-21-06673-s001.zip › ArsovicAuburger-SupplFigureS2.tif]

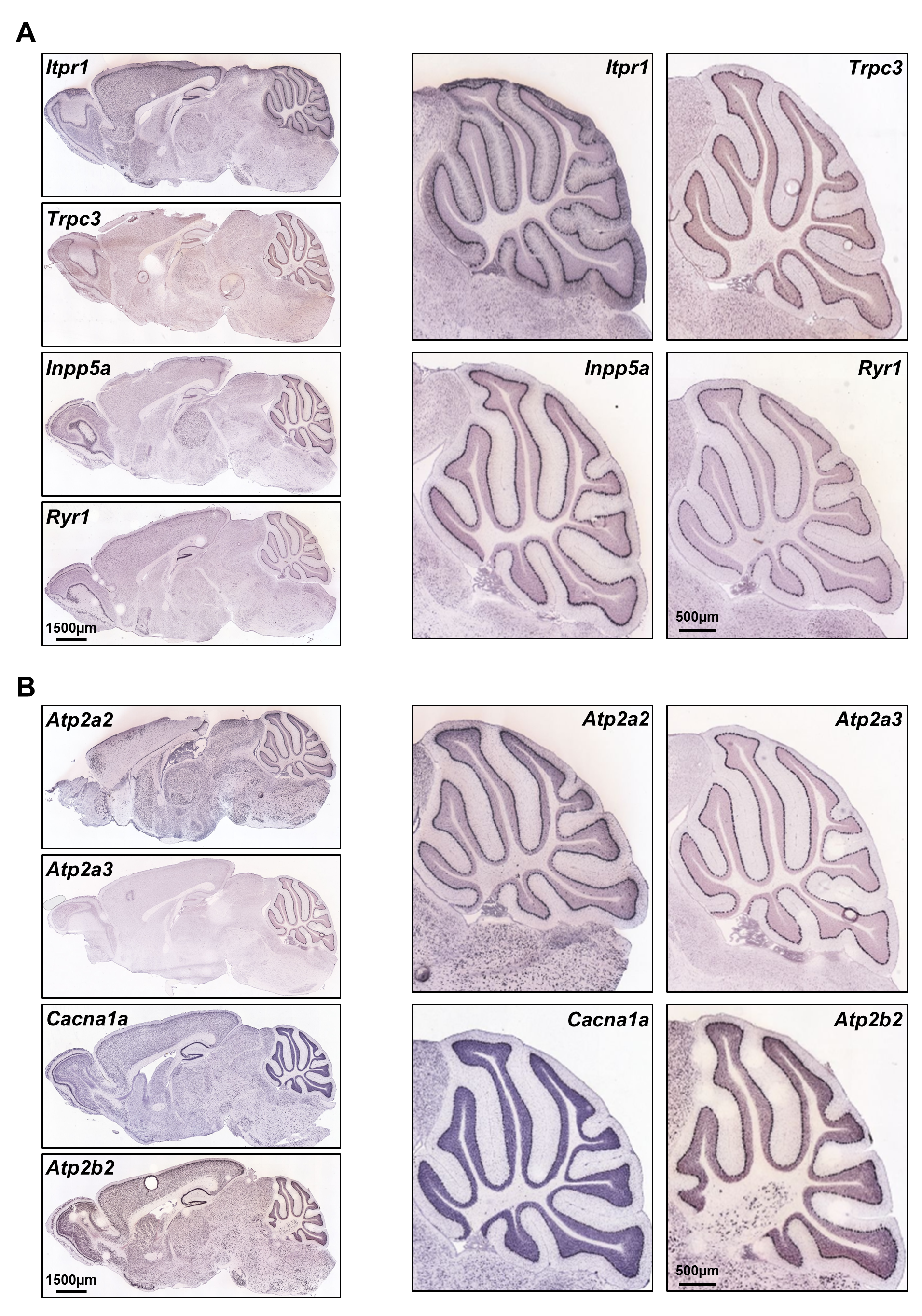

Supplement: Supplementary file 1 [file ijms-21-06673-s001.zip › ArsovicAuburger-SupplFigureS3.tif]

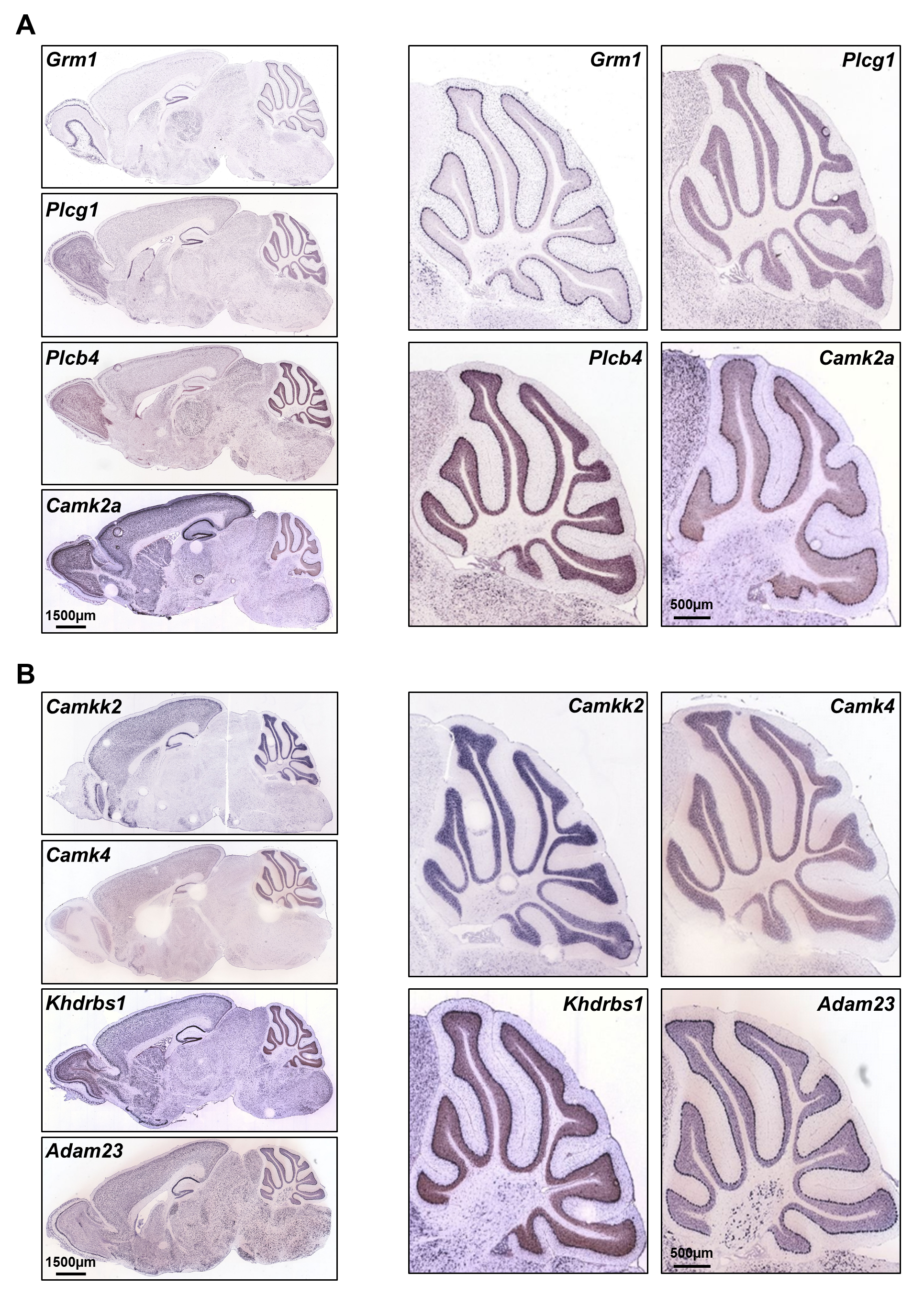

Supplement: Supplementary file 1 [file ijms-21-06673-s001.zip › ArsovicAuburger-SupplFigureS4.tif]

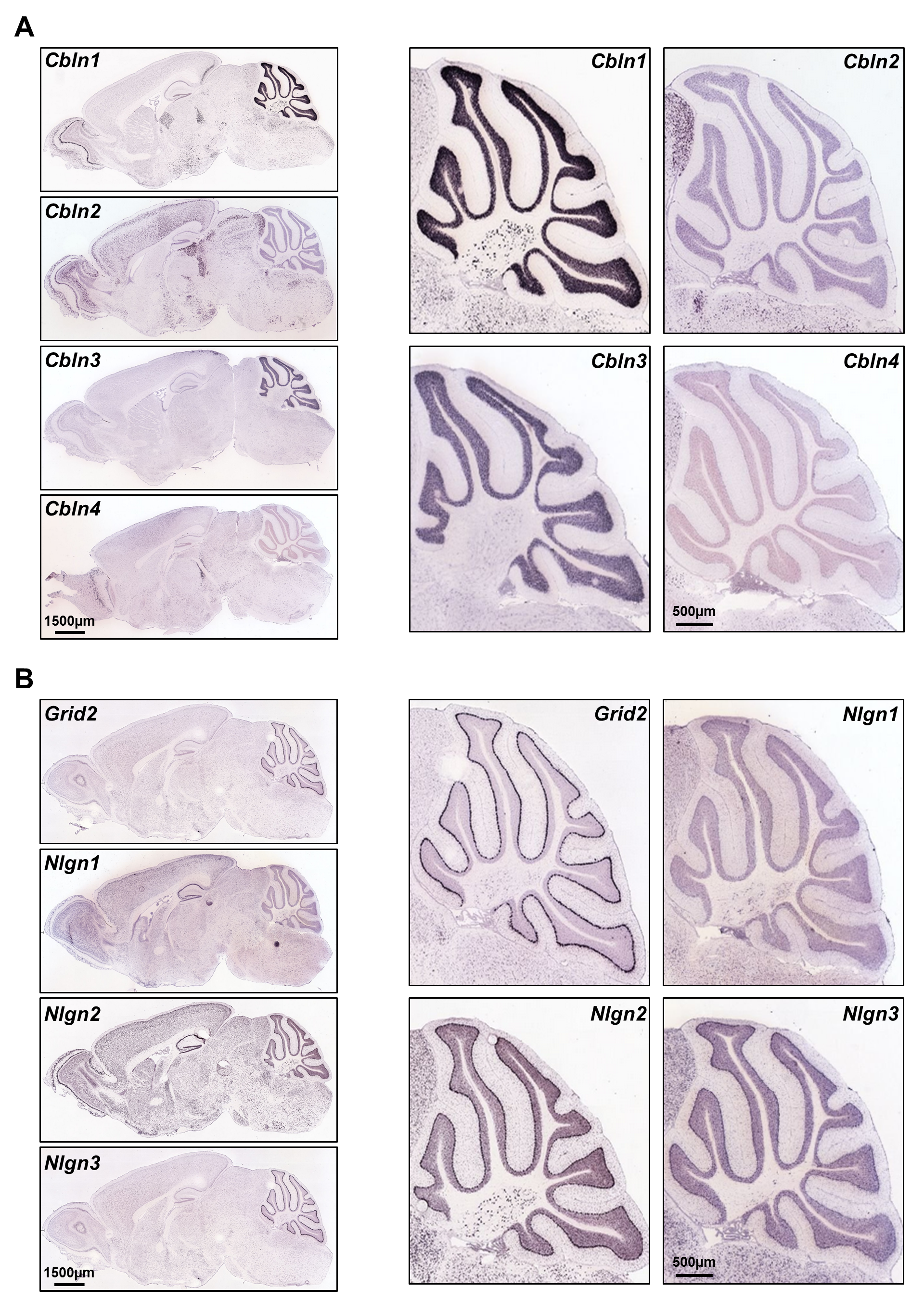

Supplement: Supplementary file 1 [file ijms-21-06673-s001.zip › ArsovicAuburger-SupplFigureS5.tif]

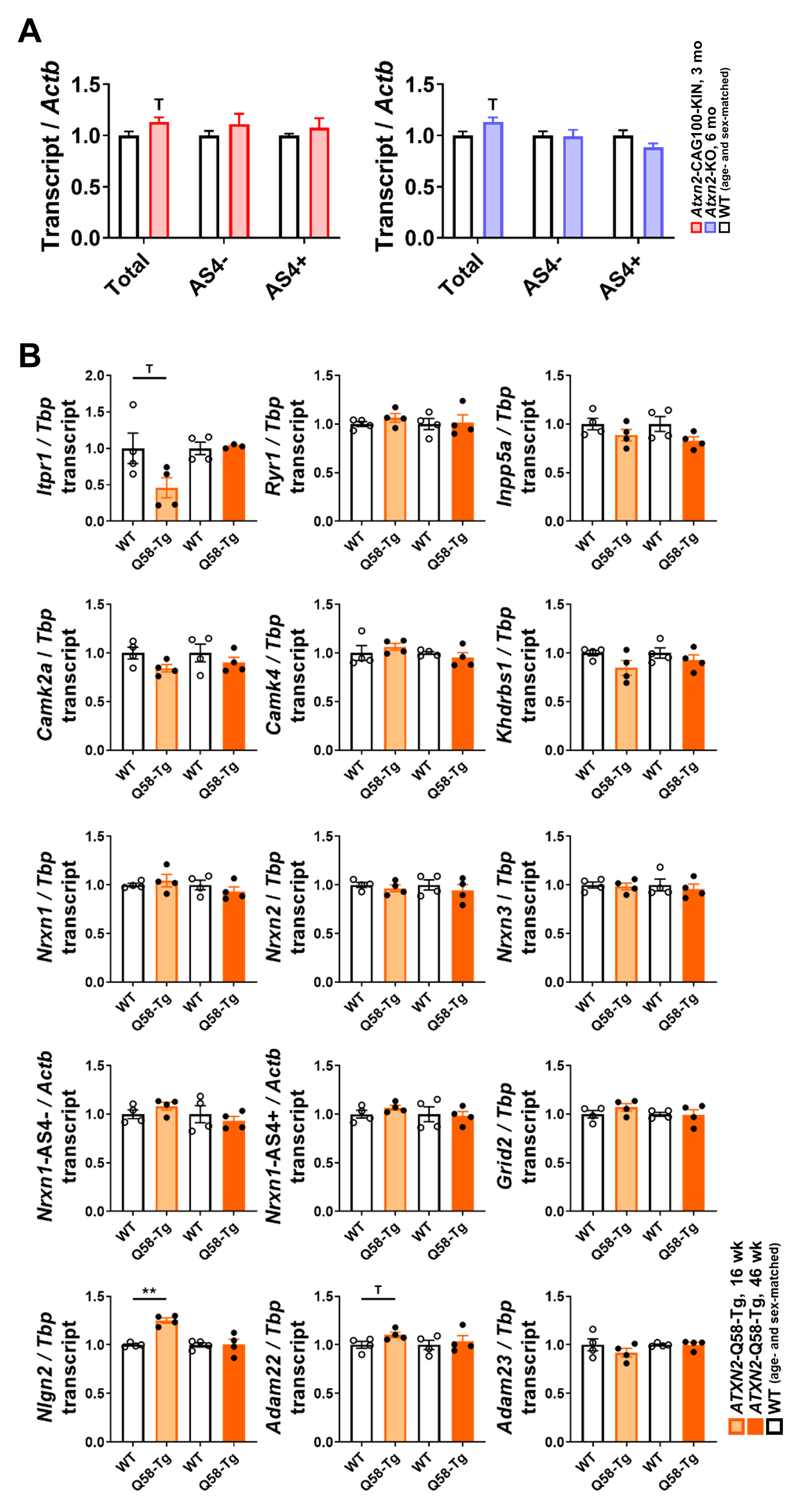

Supplement: Supplementary file 1 [file ijms-21-06673-s001.zip › ArsovicAuburger-SupplFigureS6.TIF]
